# Potent and Selective Covalent Inhibitors of the Papain-like Protease from SARS-CoV-2

**DOI:** 10.21203/rs.3.rs-906621/v1

**Published:** 2021-10-08

**Authors:** Brian Sanders, Suman Pohkrel, Audrey Labbe, Irimpan Mathews, Connor Cooper, Russell Davidson, Gwyndalyn Phillips, Kevin Weiss, Qiu Zhang, Hugh O’Neill, Manat Kaur, Lori Ferrins, Jurgen Schmidt, Walter Reichard, Surekha Surendranathan, Desigan Kumaran, Babak Andi, Gyorgy Babnigg, Nigel Moriarty, Paul Adams, Andrzej Joachimiak, Colleen Jonsson, Soichi Wakatsuki, Stephanie Galanie, Martha Head, Jerry Parks

**Affiliations:** Oak Ridge National Laboratory; Stanford University; Oak Ridge National Laboratory; SLAC National Accelerator Laboratory; Oak Ridge National Laboratory; Oak Ridge National Laboratory; Oak Ridge National Laboratory; Oak Ridge National Laboratory; Oak Ridge National Laboratory; Oak Ridge National Laboratory; Stanford University; Northeastern University; Los Alamos National Laboratory; UT Health Science Center; UT Health Science Center; Brookhaven National Laboratory; Brookhaven National Laboratory; Argonne National Laboratory; Lawrence Berkeley National Laboratory; Lawrence Berkeley Laboratory; University of Chicago; UT Health Science Center; Stanford University; Oak Ridge National Laboratory; Oak Ridge National Laboratory; Oak Ridge National Laboratory

**Keywords:** SARS-CoV-2, papain-like protease (PLpro), covalent inhibitors

## Abstract

Direct-acting antivirals for the treatment of COVID-19, which is caused by severe acute respiratory syndrome-coronavirus-2 (SARS-CoV-2), are needed to complement vaccination efforts. The papain-like protease (PLpro) of SARS-CoV-2 is essential for viral proliferation. In addition, PLpro dysregulates the host immune response by cleaving ubiquitin and interferon-stimulated gene 15 protein (ISG15) from host proteins. As a result, PLpro is a promising target for inhibition by small-molecule therapeutics. Here we have designed a series of covalent inhibitors by introducing a peptidomimetic linker and reactive electrophilic “warheads” onto analogs of the noncovalent PLpro inhibitor GRL0617. We show that the most promising PLpro inhibitor is potent and selective, with activity in cell-based antiviral assays rivaling that of the RNA-dependent RNA polymerase inhibitor remdesivir. An X-ray crystal structure of the most promising lead compound bound covalently to PLpro establishes the molecular basis for protease inhibition and selectivity against structurally similar human deubiquitinases. These findings present an opportunity for further development of potent and selective covalent PLpro inhibitors.

Coronavirus disease 2019 (COVID-19), emerged globally with the rapid spread of the previously unrecognized beta-coronavirus SARS-CoV-2.^1, 2^ The virus is highly transmissible and leads to severe, and in many cases life-threatening, respiratory disease. Efforts to repurpose existing drugs have been largely ineffective and few effective pharmaceutical treatments have been identified to date. Although vaccines are highly effective in preventing COVID-19 or reducing its severity, the emergence of variant strains may limit their effectiveness. Thus, there is an urgent need to develop new antiviral therapeutics that are effective against SARS-CoV-2 and related coronaviruses.

The SARS-CoV-2 genome encodes two cysteine proteases, 3-chymotrypsin-like protease (3CLPro) and papain-like protease (PLpro), both of which are essential for viral maturation. PLpro is a 35-kDa domain of Nsp3, a 215-kDa multidomain protein that is a key component of the viral replication complex.^3^ PLpro cleaves the viral polyproteins pp1a and pp1ab at three sites to produce nonstructural proteins Nsp1, Nsp2, and Nsp3. In addition to viral maturation, PLpro plays key roles in evading the host immune response by cleaving ubiquitin and the ubiquitin-like protein ISG15 from host protein conjugates.^4–6^ Compared to PLpro from SARS-CoV (SARS-CoV PLpro), SARS-CoV-2 PLpro displays decreased deubiquitinase (DUB) activity and enhanced deISGylation activity.^7–9^ Inhibition of SARS-CoV-2 PLpro reduces viral replication in Vero CCL-81 cells^10^ and maintains the antiviral interferon pathway.^8^

PLpro consists of thumb, fingers, and palm subdomains common to other ubiquitin-specific proteases, as well as an N-terminal ubiquitin-like domain involved in substrate recognition (Fig. 1a). The active site, which is located at the interface of the thumb and palm subdomains, consists of a catalytic triad comprising Cys111, His272, and Asp286.^10–12^ Besides the catalytic Cys111, four Cys residues coordinate a structural Zn^2+^ ion in the fingers subdomain and six additional Cys residues are present elsewhere in the protein. Of all the cysteines in PLpro, Cys111 is the most prone to oxidation,^12^ indicating that it is unique in its reactivity toward electrophiles.

Protein substrates of PLpro consist of a Leu-X-Gly-Gly peptide motif (X = Arg, Lys, or Asn) with proteolytic cleavage occurring after the second Gly residue.^4^ Leu and X occupy the S4 and S3 subsites, respectively, and the two Gly residues occupy the S2 and S1 subsites, which are covered by a b-hairpin “blocking loop” (BL2 loop) that forms a narrow groove leading to the active site (Fig. 1b).^10^ As a result, only extended peptide substrates with two Gly residues at the P1 and P2 positions can be accommodated in this space.^9, 10^

Several noncovalent inhibitors of PLpro have been developed that competitively inhibit PLpro.^12–15^ The naphthylmethylamine compound GRL0617 inhibits SARS-CoV PLpro with an IC_50_ of ~0.6 mM and inhibits viral replication in Vero E6 cells with EC_50_ = 14.5 mM.^13^ The desamino analog of GRL0617 exhibits similar inhibitory activity (IC_50_ = 2.3 μM; EC50 = 10 μM), as does the N-acetylated analog (IC_50_ = 2.6 μM; EC_50_ = 13.1 μM). GRL0617 exhibits similar inhibition activity against SARS-CoV-2 PLpro.^8, 12, 16^ Importantly, GRL0617 does not inhibit the structurally similar human DUBs. The IC_50_ values for GRL0617 toward HAUSP, the deISGylase USP18, or the ubiquitin C-terminal hydrolases UCH-L1 and UCH-L3 are all >100 mM.^13^ In addition, GRL0617 does not display cytotoxicity at concentrations up to 50 mM in cell viability assays.

We designed a series of covalent PLpro inhibitors based on the noncovalent inhibitor GRL0617 (Fig. 1 and 2). Crystal structures have revealed that the phenylmethyl group of GRL0617 points toward the active site but is located >7 Å from Sg of Cys111 (Fig. 1b). We reasoned that replacing the methyl substituent of GRL0617 with a hydrolytically stable linker connected to an electrophilic group capable of reacting with Cys111 would yield a potent covalent inhibitor of PLpro. We chose an N,N’-diacetylhydrazine linker as a linear Gly-Gly peptidomimetic that could reach through the narrow S2 and S1 groove to the active site while also preserving some of the hydrogen-bonding interactions (e.g., with Gly163 and Gly271) afforded by natural peptide substrates. To the resulting hydrazide linker we appended a series of electrophiles including a fumarate methyl ester,^17^ chloroacetamide,^18^ propiolamide, cyanoacetamide, and α-cyanoacrylamide.

To help prioritize designed molecules for synthesis and testing, we performed covalent docking of each candidate molecule to PLpro. We also docked each molecule noncovalently to assess the favorability of pre-covalent binding. We used an ensemble of 50 structural models derived from X-ray crystallographic data to account for protein flexibility^12^ and included selected crystallographic waters during docking, including those that are known to remain stably bound in the S4 subsite in the presence of noncovalent inhibitors.^12, 13^ Key interactions between PLpro and GRL0617 include (i) a hydrogen bond between the backbone N-H of Gln269 and the amide carbonyl of the inhibitor, (ii) a hydrogen bond between the N-H of the GRL0617 amide and the carboxylate side chain of Asp164, and (iii) an edge-to-face interaction of the naphthyl group of GRL0617 and Tyr268 (Fig. 1b). All candidate inhibitors contain the naphthylmethylamine core of GRL0617 (Fig. 2) and we aimed for our modified compounds to recapitulate its binding mode. To assess pose similarity, we measured the maximum common substructure RMSD (MCS-RMSD) between the docked poses of the candidate inhibitors and the crystallographic pose of GRL0617. In general, the core of the inhibitor designs reproduced the binding mode of GRL0617 to within 2 Å RMSD, maintaining interactions with Asp164, Tyr268, and Gln269 while the linker simultaneously occupied the S2 and S1 subsites to place the electrophilic group near the catalytic Cys111 nucleophile (Fig. 1e, f and Extended Data Fig. S2). Compounds were prioritized for synthesis based on low MCS-RMSD values (≤ 2 Å), favorable noncovalent and covalent docking scores (Extended Data Fig. S3 and Extended Data File 1), and synthetic tractability.

We synthesized compounds **2**-**13** and assessed their inhibition of SARS-CoV-2 PLpro using a plate-based assay with the ubiquitin C-terminus-derived Z-RLRGG-AMC fluorogenic substrate^13, 19, 20^ (Fig. 2 and Extended Data Fig. S4). IC_50_ values were determined following a 30-minute incubation of PLpro with inhibitor (Extended Data Fig. S5). Of the noncovalent analogs of GRL0617, we found that both **14** and **15** had somewhat increased IC_50_ values, with the *N*-acetylated compound **15** having an IC_50_ more like that of GRL0617. We found that extension of the methyl group with a substantially larger peptidomimetic group could maintain potency. For example, addition of the linker alone without an electrophile to form **5** led to an IC_50_ of 24 mM (Fig. 2 and Extended Data Fig. S5). The introduction of five different electrophilic warheads to produce compounds **7**, **9**, and **11–13** resulted in improved IC_50_ values for all except α-cyanoacrylamide **13**. Time-dependent inhibition assays were performed as time-dependence is consistent with multiple mechanisms of slow-binding inhibition, including covalent inhibition via bond formation between Cys111 and the electrophile. Installation of a chloroacetamide electrophile to form **9** improved the IC_50_ compared to 5 to 5.4 mM after 30-minute incubation and resulted in a *k*_*inact*_/*K*_*I*_ of 100 M^−1^ s^−1^, where *k*_*inact*_/*K*_*I*_ is a second-order rate constant describing the efficiency of the overall conversion of free enzyme to the covalent enzyme-inhibitor complex (Extended Data Fig. S6).^21^ Similarly, the IC_50_ and *k*_*inact*_/*K*_*I*_ for *N*-acetylated analog **10** are 4.4 mM and 120 M^−1^ s^−1^, respectively.

A vinyl methyl ester electrophile was recently used in tetrapeptide-based, irreversible covalent inhibitors of PLpro.^9^ We reasoned that a similar ester would occupy the oxyanion hole in the active site and engage in a hydrogen bond with Trp106. Fumarate methyl ester **7** had an IC_50_ of 94 nM after 30-minute incubation and *k*_*inact*_/*K*_*I*_ = 10,000 M^−1^ s^−1^, indicating potent inhibition (Fig. 2, 3a, b, and Extended Data Fig. S6). N-acetylated analog **8** showed similar potency, with IC_50_ and *k*_*inact*_/*K*_*I*_ = 230 nM and 14,000 M^−1^ s^−1^, respectively. To examine the inhibitory activity of other electrophiles, we synthesized and performed time-independent inhibition assays with cyanoacetamide **11** (IC_50_, 8 mM), propiolamide **12** (98 nM), and a-cyanoacrylamide **13** (>200 mM). Time-dependent inhibition was observed for **12**, but not for **11** or **13** (Extended Data Fig. S7). To provide additional evidence for a covalent mechanism of action, compounds **7–10** and **12** were incubated with PLpro, and the protein intact masses were determined by electrospray ionization mass spectrometry (ESI-MS). Covalent adduct formation with PLpro was confirmed for these five compounds (Fig. 3c, Extended Data Fig. S8 and Extended Data Table 1).

Following the promising results from in vitro assays and mass spectrometry experiments, we used X-ray crystallography to obtain structural insight into covalent inhibition of PLpro. We determined a crystal structure of wild-type PLpro in complex with fumarate methyl ester **7**, the most promising lead, at 3.10 Å resolution (Extended Data Table S2). The electron density maps show clear densities for PLpro, Zn cations, and **7**, confirming the design concept of this compound and revealing key interactions with PLpro (Fig. 4). A covalent bond is present between Sg of Cys111 and C1 of compound **7** (Fig. 4a). The carbonyl oxygen from the fumarate ester accepts hydrogen bonds from the indole side chain of Trp106, like that of the tetrapeptide-based covalent inhibitor VIR251,^9^ as well as the side chain of Asn109. The N, N’-diacetylhydrazine moiety was designed to link the electrophile and the naphthylmethylamine core while also hydrogen bonding with residues in the S1-S2 groove. Indeed, the crystal structure revealed that the proximal and distal carbonyl oxygens of the N, N’-diacetylhydrazine linker interact with the backbone N-H groups of Gly163 and Gly271, and the proximal and distal N-H groups of this moiety participate in hydrogen bonds with the carbonyl backbones of Gly271 and Gly163. As intended, the carbonyl oxygen and N-H group of the amide adjacent to the naphthyl group of **7** are hydrogen bonded with the N-H backbone of Gln269 and the carboxylate side chain of Asp164. Compound **7** makes five main-chain and three side-chain hydrogen bonding interactions in the binding site. In addition, the side chains of Tyr268 and Gln269 interact with **7** similarly to GRL0617. Electron density for the methyl group of the ester of **7** was not visible. It is possible that the ester linkage is flexible and adopts multiple conformations or that it could have been hydrolyzed. Encouragingly, the covalently docked pose for **7** agrees closely with the co-crystal structure (Fig. 4b).

The ability of the inhibitors to protect Vero E6 cells from viral infection-induced cell death, represented by EC_50_ (Fig. 2, 3d and Extended Data Fig. S9), was assessed by incubating cells with and without compound and then infecting them with SARS-CoV-2.^25^ Uninfected cells were used to assess the cytotoxicity of the compounds, represented by CC_50_ (Fig. 2). Compound **7** displayed notable antiviral activity with an EC_50_ of 1.1 μM, comparable to that of the remdesivir drug control (0.74 μM). Chloroacetamide **9** also displayed antiviral activity, although with less potency (34 mM). Neither **7** nor **9** displayed evidence of cytotoxicity (CC_50_ > 30 mM). Compounds **8** and **10**, which have N-acetylated phenyl substituents, showed insignificant cytoprotective effects. Both **12** and **13** were cytotoxic with CC_50_ values of 1–5 μM, suggesting that propiolamide and α-cyanoacrylamide electrophiles may be too reactive, lack specificity, or both.

In addition to its role in processing the replicase polyprotein, SARS-CoV-2 PLpro displays deubiquitinase and de-ISG15ylase activity.^10, 26^ To ensure that our most promising covalent inhibitors **7** and **9** can inhibit these physiologically relevant activities, IC_50_ values were obtained with Ub-rhodamine and ISG15-CHOP2 substrates (Extended Data File 2). Compound **7** inhibited PLpro with Ub-rhodamine and ISG15 substrates with IC_50_ values of 76 and 39 nM, respectively. The corresponding IC_50_ values for **9** with these two substrates were 1.96 μM and 20.2 μM, respectively.

Because PLpro bears structural and functional similarity to human DUBs and related enzymes, inhibitor selectivity is an important consideration. Seven human DUBs, UCHL1, USP2, USP4, USP7, USP8, USP15, and USP30, were assayed to determine whether they were inhibited by **7** and **9**. No inhibition of the human DUBs was observed for either compound at concentrations up to 30 μM (Extended Data File 2). In silico analysis of the superposed structures of human DUBs with the active site residues and the helix bearing Cys111 of the co-crystal structure of PLpro with **7** suggests that the naphthyl ring in **7** would experience severe clashes with the crossover loop (Arg153-Lys157) of UCHL1, and Phe828 and Lys838 of USP4 (Fig. 4d and 4e, respectively), providing a structural basis for the selectivity of **7** against human DUBs. Thus, compounds **7** and **9** inhibit PLpro-mediated peptide cleavage, ubiquitin cleavage, and ISG15 cleavage, they have antiviral activity against SARS-CoV-2 and lack cytotoxicity in Vero E6 cells, and they do not inhibit a representative panel of human DUBs.

We next sought to determine the metabolic stability of our compounds in human, rat, and mouse liver microsomes and the corresponding S9 fractions (Extended Data Table S3 and S4). Chloroacetamide **9** demonstrated very short half-lives of 3 and 7 minutes in human liver S9 and microsomes, respectively, likely due to the highly reactive electrophile. Non-covalent inhibitor **14** exhibited a half-life >60 min in the S9 fraction, and 41 min in microsomes. Conversion to its covalent counterpart **7** maintained the half-life (60 min in S9, 50 min in microsomes). Analysis of **14** and **7** with MetaSite 6.0.1^27^ suggested that successive oxidations of the tolyl methyl of **14** were the predominant metabolic liability, followed by the benzylic methylene (Extended Data Figure S10). Given that the linker and electrophile replaced the labile methyl group, it is unsurprising that the benzylic methylene is predicted to be the primary site of metabolism for **7**. To address the benzylic liability several modifications could be pursued, including substitution of the benzylic position with heavy atoms such as deuterium^29^ or fluorine^30^ to increase steric hindrance,^28^ or blocking the site of metabolism via replacement of the tolyl methyl with cyclopropane.^31^

Numerous research efforts have focused on developing inhibitors of 3CLpro, but relatively few have focused on PLpro inhibition. A predominant reason for the emphasis on 3CLpro as an antiviral target is that there are no structural homologs in the human proteome whereas PLpro bears structural similarity to human DUBs and deISGylases. However, our findings demonstrate that covalent inhibition of PLpro is a promising strategy for developing potent and selective therapeutics to combat SARS-CoV-2. Furthermore, a crystal structure of our most promising inhibitor covalently bound to PLpro provides insight that will facilitate the development of next-generation PLpro inhibitors with enhanced pharmacokinetic and pharmacodynamic properties.

## Methods

### Docking preparation.

The 2.09 Å X-ray co-crystal structure of the C111S mutant of PLpro with GRL0617 (PDB entry 7JIR)^12^ was used for the docking calculations. Rather than docking to a single structure, we used PHENIX^32^ to generate an ensemble^33^ of 50 conformations from the corresponding crystallographic data in which conformations were sampled to generate an ensemble that collectively fit the data better than any single model. This approach provides valuable information about regions of high and low conformational variability in the protein, such as the BL2 loop, which is known to undergo large conformational changes upon substrate or inhibitor binding. Ser111 was converted back to Cys in all models.

Selected water molecules present in the models were retained during docking. Cys111 was modeled as a neutral thiol and His272 was protonated on Ne in accordance with its local hydrogen bonding environment and the proton transfer chemistry that is expected to occur during catalysis. Other histidines were protonated based on their inferred hydrogen bonding patterns. All other residues were protonated according to their canonical pH 7.0 protonation states. The program *tleap* from AmberTools20^34^ was used to prepare the parameter and coordinate files for each structure. The ff14SB force field^35^ and TIP3P water model^36^ were used to describe the protein and solvent, respectively. Energy minimization was performed using *sander* from AmberTools20 with 500 steps of steepest descent, followed by 2000 steps of conjugate gradient minimization. Harmonic restraints with force constants of 200 kcal mol^−1^ Å^−1^ were applied to all heavy atoms during energy minimization.

The peptide substrate binding cleft of PLpro spans ∼30 Å along the interface of the palm and thumb domains (Extended Data Fig. S1). Thus, we defined a rectangular docking box spanning the entire binding cleft (S1-S4 sites) and the active site (catalytic triad). AutoGrid Flexible Receptor (AGFR)^37^ was used to generate the receptor files for both noncovalent and covalent docking using a grid spacing of 0.25 Å. All docking calculations were performed with AutoDock Flexible Receptor (ADFR).^37^ Compounds with electrophilic groups were docked both noncovalently (i.e., in the reactive form with an explicit electrophile present) and covalently (i.e., in the post-reactive Cys111 adduct form).

### Ligand preparation.

SMILES strings for candidate inhibitor designs were converted to PDB format using Open Babel^38^ and custom Python/RDKit^39^ scripts. Covalent docking with AutoDockFR requires that ligands be modified such that they include the covalent linkage to the side chain of the reactive residue, in this case Cys111, which then serves as an anchor to place the ligand approximately in the binding site.^37^ Thus, the Ca and Cb atoms of Cys111 were used as anchors and the backbone N atom of Cys111 was used to define a torsional angle connecting the covalently bound ligand and the protein. MGLTools 1.5.6^40^ was used to generate PDBQT files for ligands and receptors. Only polar hydrogens were retained during docking.

All candidate inhibitors considered in this work include the naphthylmethylamine core of GRL0617, for which co-crystal structures are available.^12^ We expected that our covalent compounds would adopt a pose like GRL0617. Thus, to assess the similarity between the poses of docked candidate ligands and GRL0617 in the X-ray structure, we calculated the maximum common substructure (MCS) RMSD between them. MCS RMSDs were calculated for poses with docking energies within 3 kcal/mol of the overall most favorable pose for each candidate inhibitor. Compounds were prioritized for synthesis that had docked poses with MCS-RMSD values ≤2 Å and favorable noncovalent and covalent docking scores (Extended Data Fig. S2 and Extended Data File 1). Figures were generated with PyMOL.^41^

### Synthesis and Characterization of Compounds.

All reagents were purchased from commercial suppliers and used as received unless otherwise noted. Anhydrous acetonitrile (MeCN), dichloromethane (CH_2_Cl_2_), ethanol (EtOH), dimethylformamide (DMF), tetrahydrofuran (THF), methanol (MeOH), and diethyl ether (Et_2_O) were purchased from commercial sources and maintained under dry N_2_ conditions. Amide couplings and reactions with acid chlorides were performed under N_2_ using standard Schlenk-line techniques. Compound **1** was purchased from commercial sources and used as received. ^1^H and ^13^C NMR spectra were recorded in the listed deuterated solvent with either Bruker Avance III HD 500 MHz NMR spectrometer at 298 K with chemical shifts referenced to the residual protio signal of the deuterated solvent as previously reported.^42^ Mass data were collected on a Waters Synapt HDMS QTOF mass spectrometer.

#### 5-acetamido-2-(3-methoxy-3-oxopropyl)benzoic acid (2).

To a 15 mL solution of DCM was added 0.300 g (1.344 mmol) of 5-amino-2-(3-methoxy-3-oxopropyl)benzoic acid and cooled to 0 °C. Acetic anhydride (1.3 mL, ~13 mmol) was added slowly while stirring. The solution was allowed to reach RT overnight, followed by addition of saturated NH_4_Cl and extraction with DCM (3 × 50 mL). The organic phases were combined and dried with MgSO_4_ and concentrated under reduced pressure to afford a pale-yellow syrup (0.195 g, 0.735 mmol, 55%). ^1^H NMR (500 MHz, DMSO-*d*_6_, δ from residual protio solvent) δ 12.40 (s, br, 1H), 10.00 (s, 1H), 8.03 (s, 1H), 7.67 (d, *J* = 8.3 Hz, 1H), 7.23 (d, *J* = 8.3 Hz, 1H), 3.57 (s, 3H), 3.10 (t, *J* = 7.7 Hz, 2H), 2.56 (t, *J* = 7.7 Hz, 2H), 2.03 (s, 3H). ^13^C NMR (126 MHz, DMSO, δ from solvent) δ 172.61, 168.32, 137.54, 135.83, 131.09, 130.43, 122.18, 120.75, 51.18, 35.08, 28.50, 23.88, 20.99. LRMS-ESI (*m/z*): [M + H]^+^ Theoretical for C_13_H_15_NO_5_: 266.1; Experimental: 266.1.

#### methyl (*R*)-3-(2-((1-(naphthalen-1-yl)ethyl)carbamoyl)phenyl)propanoate (3).

A 20 mL DCM solution containing 2-(3-methoxy-3-oxopropyl)benzoic acid (0.500 g, 2.4 mmol) was cooled to 0 °C followed by addition of HBTU (1.138 g, 3.0 mmol). This solution was stirred for 30 min, followed by addition of (*R*)-1-(naphthalen-1-yl)ethan-1-amine (0.409 g, 2.4 mmol) and DIPEA (0.522 mL, 3.0 mmol). The solution was warmed to RT and stirred for 16 h. The reaction mixture was quenched with 50 mL of H_2_O and extracted with DCM (3×50 mL). The organic layers were collected and dried with MgSO_4_ and concentrated under reduced pressure. The residue was purified by silica gel chromatography using 3:1 Hexanes:EtOAc (*R*_f_ = 0.36) to afford a white solid. Washes were performed, and the resulting solid was dried under reduced pressure. This workup afforded the product as an off-white solid (0.723 g, 2.0 mmol, 83%). ^1^H NMR (500 MHz, DMSO-*d*_6_) δ from residual protio solvent 8.95 (d, *J* = 7.9 Hz, 1H), 8.24 (d, *J* = 8.4 Hz, 1H), 7.95 (d, *J* = 8.0 Hz, 1H), 7.84 (d, *J* = 8.1 Hz, 1H), 7.65 – 7.46 (m, 4H), 7.38 – 7.29 (m, 2H), 7.30 – 7.23 (m, 2H), 5.92 (p, *J* = 7.2 Hz, 1H), 3.57 (s, 3H), 2.92 (t, *J* = 8.0 Hz, 2H), 2.57 (t, *J* = 7.9 Hz, 2H), 1.58 (d, *J* = 6.9 Hz, 3H). ^13^C NMR (126 MHz, DMSO, δ from solvent): 172.51, 168.02, 140.12, 138.11, 136.96, 133.36, 130.39, 129.56, 129.34, 128.62, 127.29, 127.19, 126.11, 126.00, 125.56, 125.43, 123.11, 122.46, 51.21, 44.36, 34.96, 27.96, 21.36. HRMS-ESI (*m/z*): [M + H]^+^ Theoretical for C_23_H_24_NO_3_: 362.1756; Experimental: 362.1745.

#### methyl (*R*)-3-(4-acetamido-2-((1-(naphthalen-1-yl)ethyl)carbamoyl)phenyl)propanoate (4).

Compound **4** was prepared similarly to the amide coupling of **3**. The amount of materials used were: **2** (0.350 g, 1.08 mmol); HBTU (0.899 g, 2.15 mmol); (*R*)-1-(naphthalen-1-yl)ethan-1-amine (0.366 g, 2.15 mmol) and DIPEA (0.749 mL, 4.30 mmol). Silica gel column purification was performed under a gradient from 1:1, 2:1, 3:1 EtOAc:Hexanes at 1 column volume for each gradient step. Compound **4** was isolated as white solid (0.410 g, 0.980 mmol, 91%). ^1^H NMR (500 MHz, DMSO-*d*_6_, δ from residual protio solvent) δ 9.96 (s, 1H), 8.95 (d, *J* = 8.0 Hz, 1H), 8.24 (d, *J* = 8.4 Hz, 1H), 7.95 (dd, *J* = 8.0, 1.6 Hz, 1H), 7.84 (d, *J* = 8.2 Hz, 1H), 7.64 – 7.55 (m, 3H), 7.54 (ddd, *J* = 8.1, 6.8, 1.3 Hz, 1H), 7.52 – 7.45 (m, 2H), 7.17 (d, *J* = 8.4 Hz, 1H), 5.92 (p, *J* = 7.2 Hz, 1H), 3.56 (s, 3H), 2.83 (t, *J* = 7.8 Hz, 2H), 2.69 (s, 3H), 2.53 (t, *J* = 8.0 Hz, 2H), 2.02 (s, 3H), 1.57 (d, *J* = 6.9 Hz, 3H). ^13^C NMR (126 MHz, DMSO, δ from solvent) δ 172.50, 168.22, 167.88, 140.07, 137.33, 137.26, 133.33, 132.26, 130.39, 129.78, 128.60, 127.19, 126.14, 125.56, 125.36, 123.08, 122.39, 119.69, 117.71, 51.17, 44.22, 38.19, 35.02, 27.39, 23.85, 21.39. LRMS-ESI (*m/z*): [M + H]^+^ Theoretical for C_25_H_26_N_2_O_4_: 419.2; Experimental: 419.2.

#### (*R*)-2-(3-hydrazineyl-3-oxopropyl)-*N*-(1-(naphthalen-1-yl)ethyl)benzamide (5).

To a 10 mL EtOH solution containing **1** (0.400 g, 1.11 mmol) was added 0.5 mL (~1 M) of hydrazine monohydrate (N_2_H_4_ 64–65%, reagent grade 95%). The pale-yellow, homogenous solution was refluxed for 16 h. The resulting solution was reduced under vacuum to afford an off-white powder. To remove excess hydrazine monohydrate, several (3×15 mL) Et_2_O washes were performed, and the resulting solid was dried under reduced pressure. This workup afforded the product as an off-white solid (0.390 g, 1.08 mmol, 97%). ^1^H NMR (500 MHz, DMSO-*d*_6_, δ from residual protio solvent): 8.97 (d, *J* = 7.9 Hz, 1H), 8.91 (s, 1H), 8.25 (d, *J* = 8.5 Hz, 1H), 7.96 (d, *J* = 8.1 Hz, 1H), 7.84 (d, *J* = 8.1 Hz, 1H), 7.65 (d, *J* = 7.2 Hz, 1H), 7.61 (t, *J* = 7.6 Hz, 1H), 7.54 (dt, *J* = 15.0, 7.6 Hz, 2H), 7.35 (t, *J* = 7.4 Hz, 1H), 7.31 (d, *J* = 7.4 Hz, 1H), 7.28 – 7.21 (br, 2H), 5.93 (p, *J* = 7.2 Hz, 1H), 4.21 (s, 2H), 2.91 (td, *J* = 7.5, 4.3 Hz, 2H), 2.35 (t, *J* = 7.9 Hz, 2H), 1.60 (d, *J* = 6.9 Hz, 3H). ^13^C NMR (126 MHz, DMSO, δ from solvent): 170.82, 168.04, 140.20, 138.74, 137.05, 133.35, 130.37, 129.22, 129.20, 128.61, 127.21, 127.16, 126.14, 125.72, 125.55, 125.50, 123.12, 122.46, 44.42, 34.85, 28.22, 21.44. HRMS-ESI (*m/z*): [M + H]^+^ Theoretical for C_22_H_24_N_3_O_2_: 362.1859; Experimental: 362.1885.

#### (*R*)-5-acetamido-2-(3-hydrazineyl-3-oxopropyl)-*N*-(1-(naphthalen-1-yl)ethyl)benzamide (6).

Compound **6** was prepared analogously to **5**. The amounts of materials used were: **4** (0.400 g, 0.956 mmol); 10 mL EtOH solution containing; 0.5 mL (~1M) of hydrazine monohydrate (N_2_H_4_ 64–65%, reagent grade 95%). ^1^H NMR (500 MHz, DMSO-*d*_6_, δ from residual protio solvent) δ 9.94 (s, 1H), 8.97 (d, *J* = 7.9 Hz, 1H), 8.89 (s, 1H), 8.24 (d, *J* = 8.4 Hz, 1H), 7.95 (d, *J* = 8.1 Hz, 1H), 7.84 (d, *J* = 8.0 Hz, 1H), 7.65 – 7.56 (m, 3H), 7.53 (dt, *J* = 18.1, 7.5 Hz, 2H), 7.45 (s, 1H), 7.15 (d, *J* = 8.4 Hz, 1H), 5.92 (p, *J* = 6.9 Hz, 1H), 4.11 (s, br, 2H), 2.82 (hept, *J* = 7.5, 7.0 Hz, 2H), 2.31 (t, 2H), 2.01 (s, 3H), 1.58 (d, *J* = 7.0 Hz, 3H). ^13^C NMR (126 MHz, DMSO, δ from solvent) δ 170.85, 168.18, 167.90, 140.17, 137.40, 137.02, 133.34, 132.89, 130.39, 129.42, 128.60, 127.18, 126.17, 125.57, 125.44, 123.11, 122.39, 119.68, 117.66, 44.31, 34.89, 27.65, 23.85, 21.48. LRMS-ESI (*m/z*): [M + H]^+^ Theoretical for C_25_H_26_N_4_O_3_: 419.2; Experimental: 419.2.

#### Preparation of compounds with electrophilic warheads.

Compounds **7**, **9**, **11**, and **13** were prepared by taking 0.030 g (0.083 mmol) of **5** and 0.029 mL (0.166 mmol) of DIPEA into 5 mL anhydrous DCM under N_2_ atmosphere. Once dissolved, 0.100 mmol (1.2 equiv.) of appropriate acid chloride was added while stirring under N_2_ atmosphere. Rapid reaction resulted in precipitation of a white solid. The reaction was left at RT for 2 h with no observable changes. The DCM was removed under reduced pressure and Et_2_O was added to the remaining residue to precipitate a white solid that was collected with a 2 mL fritted glass funnel. The remaining white solid was washed extensively with Et_2_O, dried, and collected. Isolated yields: **7** (0.022 g, 0.046 mmol, 56%); **9** (0.018 g, 0.041 mmol, 50%); **11** (0.020 g, 0.047 mmol, 56%); **13** (0.024 g, 0.050 mmol, 60%).

Compounds **8** and **10** were prepared by placing 0.040 g (0.096 mmol) of **6** in 5 mL of anhydrous DMF followed by addition of K_2_CO_3_ (0.020 g, 0.145 mmol). The solution was stirred while 0.115 mmol (1.2 equiv.) of appropriate acid chloride was added. The solution was stirred at RT for 2 h followed by addition of 25 mL EtOAc and extraction with 3×25 mL of H_2_O to remove DMF. The organic layers were combined, dried with MgSO_4_, and concentrated under reduced pressure. The crude residue was purified by silica gel flash chromatography using pure EtOAc with 1–5% MeOH to yield white solids: **8** (0.016 g, 0.032 mmol, 34%); **10** (0.019 g, 0.036 mmol, 37%).

#### methyl(*R,E*)-4-(2-(3-(2-((1-(naphthalen-1 yl)ethyl)carbamoyl)phenyl)propanoyl)hydrazineyl)-4-oxobut-2-enoate (7).

^1^H NMR (500 MHz, DMSO-*d*_6_, δ from residual protio solvent) δ 10.53 (s, 1H), 10.16 (s, 1H), 8.93 (d, *J* = 7.9 Hz, 1H), 8.24 (d, *J* = 8.6 Hz, 1H), 7.95 (d, *J* = 8.1 Hz, 1H), 7.83 (d, *J* = 8.2 Hz, 1H), 7.67 – 7.57 (m, 2H), 7.56 – 7.48 (m, 2H), 7.39 – 7.21 (m, 4H), 7.07 (d, *J* = 15.6 Hz, 1H), 6.68 (d, *J* = 15.5, 1H), 5.93 (p, *J* = 7.3 Hz, 1H), 3.75 (s, 3H), 2.99 – 2.89 (m, 2H), 2.52 (m, 2H), 1.59 (d, *J* = 6.9 Hz, 3H). ^13^C NMR (126 MHz, DMSO, δ from solvent) δ 170.30, 168.56, 165.73, 161.57, 140.66, 139.06, 137.56, 135.58, 133.86, 130.91, 129.90, 129.82, 129.77, 129.13, 127.73, 127.70, 126.67, 126.35, 126.08, 126.02, 123.65, 122.96, 52.59, 44.94, 35.14, 28.56, 21.92. HRMS-ESI (*m/z*): [M + H]^+^ Theoretical for C_27_H_28_N_3_O_5_: 474.2029; Experimental: 474.2007.

#### methyl (*R,E*)-4-(2-(3-(4-acetamido-2-((1-(naphthalen-1-yl)ethyl)carbamoyl)phenyl)propanoyl)hydrazineyl)-4-oxobut-2-enoate (8).

^1^H NMR (500 MHz, DMSO-*d*_6_, δ from residual protio solvent) δ 10.52 (s, 1H), 10.15 (s, 1H), 9.95 (s, 1H), 8.93 (d, *J* = 7.9 Hz, 1H), 8.24 (d, *J* = 8.6 Hz, 1H), 7.95 (d, *J* = 8.0 Hz, 1H), 7.83 (d, *J* = 8.2 Hz, 1H), 7.64 – 7.57 (m, 3H, 7.56 – 7.48 (m, 2H), 7.45 (s, 1H), 7.21 (d, *J* = 8.4 Hz, 1H), 7.07 (d, *J* = 15.6 Hz, 1H), 6.68 (d, *J* = 15.5 Hz, 1H), 5.93 (p, *J* = 7.2 Hz, 1H), 3.75 (s, 3H), 2.86 (m, 2H), 2.47 (m, 2H), 2.02 (s, 3H), 1.57 (d, *J* = 6.9 Hz, 3H). ^13^C NMR (126 MHz, DMSO, δ from solvent) δ 169.79, 168.17, 167.88, 165.18, 161.03, 140.09, 137.38, 137.09, 135.02, 133.31, 132.65, 130.37, 129.57, 129.22, 128.57, 127.17, 126.16, 125.55, 125.41, 123.09, 122.34, 119.68, 117.60, 52.04, 44.27, 34.64, 27.45, 23.83, 21.41. HRMS-ESI (*m/z*): [M + H]^+^ Theoretical for C_29_H_31_N_4_O_6_: 531.2244; Experimental: 531.2217.

#### (*R*)-2-(3-(2-(2-chloroacetyl)hydrazineyl)-3-oxopropyl)-*N*-(1-(naphthalen-1-yl)ethyl)benzamide (9).

^1^H NMR (500 MHz, DMSO-*d*_6_, δ from residual protio solvent) δ 10.21 (s, 1H), 9.98 (s, 1H), 8.95 (d, *J* = 7.8 Hz, 1H), 8.24 (d, *J* = 8.5 Hz, 1H), 7.96 (d, *J* = 8.1 Hz, 1H), 7.84 (d, *J* = 8.1 Hz, 1H), 7.67 – 7.49 (m, 4H), 7.38 – 7.23 (m, 4H), 5.93 (p, *J* = 7.2 Hz, 1H), 4.14 (s, 2H), 2.94 (t, *J* = 9.1, 2H), 2.48 (t, *J* = 9.1 Hz, 2H), 1.60 (d, *J* = 6.8 Hz, 3H). ^13^C NMR (126 MHz, DMSO, δ from solvent) δ 170.08, 168.06, 164.65, 140.15, 138.56, 137.04, 133.35, 130.39, 129.39, 129.31, 128.62, 127.20 (two overlapping ^13^C signals), 126.16, 125.83, 125.58, 125.51, 123.14, 122.45, 44.43, 40.86, 34.62, 28.02, 21.41 HRMS-ESI (*m/z*): [M + H]^+^ Theoretical for C_24_H_25_ClN_3_O_3_: 438.1584; Experimental: 438.1565.

#### (*R*)-5-acetamido-2-(3-(2-(2-chloroacetyl)hydrazineyl)-3-oxopropyl)-*N*-(1-(naphthalen-1-yl)ethyl)benzamide (10).

^1^H NMR (500 MHz, DMSO-*d*_6_, δ from residual protio solvent) δ 10.20 (s, 1H), 9.96 (s, 2H), 8.94 (d, *J* = 8.0 Hz, 1H), 8.25 (d, *J* = 8.5 Hz, 1H), 7.96 (d, *J* = 8.1 Hz, 1H), 7.85 (d, *J* = 8.2 Hz, 1H), 7.62 (q, *J* = 6.7 Hz, 3H), 7.54 (m, 2H), 7.46 (s, 1H), 7.21 (d, *J* = 8.4 Hz, 1H), 5.93 (q, *J* = 7.3 Hz, 1H), 4.14 (s, 2H), 2.86 (m, 2H), 2.45 (t, *J* = 7.9 Hz, 2H), 2.03 (s, 3H), 1.58 (d, *J* = 6.8 Hz, 3H). ^13^C NMR (126 MHz, DMSO, δ from solvent) δ 170.60, 168.72, 168.43, 165.14, 140.64, 137.92, 137.63, 133.86, 133.21, 130.92, 130.12, 129.12, 127.72, 126.71, 126.10, 125.97, 123.64, 122.89, 120.23, 118.13, 44.82, 41.37, 35.18, 27.97, 24.38, 21.96. HRMS-ESI (*m/z*): [M + H]^+^ Theoretical for C_26_H_28_ClN_4_O_4_: 495.1799; Experimental: 495.1788.

#### (*R*)-2-(3-(2-(2-cyanoacetyl)hydrazineyl)-3-oxopropyl)-*N*-(1-(naphthalen-1-yl)ethyl)benzamide (11).

^1^H NMR (500 MHz, DMSO-*d*_6_, δ from residual protio solvent) δ 10.16 (s, 1H), 9.96 (s, 1H), 8.93 (d, *J* = 7.8 Hz, 1H), 8.23 (d, *J* = 8.5 Hz, 1H), 7.95 (d, *J* = 8.2 Hz, 1H), 7.83 (d, *J* = 8.2 Hz, 1H), 7.66 – 7.57 (m, 2H), 7.57 – 7.48 (m, 2H), 7.39 – 7.21 (m, 4H), 5.92 (p, *J* = 7.1 Hz, 1H), 3.74 (s, 2H), 2.97 – 2.89 (t, 7.6 Hz, 2H), 2.47 (t, *J* = 7.6 Hz, 2H), 1.59 (d, *J* = 6.9 Hz, 3H). ^13^C NMR (126 MHz, DMSO, δ from solvent) δ 170.13, 168.03, 161.12, 140.14, 138.52, 137.02, 133.34, 130.39, 129.39, 129.29, 128.61, 127.20, 127.18, 126.15, 125.82, 125.57, 125.50, 123.13, 122.44, 115.62, 44.41, 34.55, 27.99, 23.67, 21.39. HRMS-ESI (*m/z*): [M + H]^+^ Theoretical for C_25_H_25_N_4_O_3_: 429.1928; Experimental: 429.1949.

#### (*R*)-*N*-(1-(naphthalen-1-yl)ethyl)-2-(3-oxo-3-(2-propioloylhydrazineyl)propyl)benzamide (12).

Compound **12** was synthesized under the same conditions as compounds **7**, **9**, **11**, and **13** except the initial coupling to the hydrazide of **5** was achieved with 3-(trimethylsilyl)propioloyl chloride. The DCM was removed under reduced pressure and the crude material was immediately dissolved in 1:1 THF:MeOH (6 mL total volume) and 10 mg of K_2_CO_3_ was added. The solution was stirred and monitored by TLC until the reaction was complete, approximately 30 min. The solution was concentrated and purified by silica gel flash chromatography (2:1 EtOAc:Hexanes) to yield 8 mg (0.019 mmol, 23%) of a pale yellow solid. ^1^H NMR (500 MHz, Acetone-*d*_6_, δ from residual protio solvent) δ 9.49 (s, 1H), 9.12 (s, 1H), 8.33 (d, *J* = 8.6 Hz, 1H), 8.05 (d, *J* = 8.2 Hz, 1H), 7.93 (d, *J* = 8.2 Hz, 1H), 7.83 (d, *J* = 8.2 Hz, 1H), 7.72 (d, *J* = 7.2 Hz, 1H), 7.63 (t, *J* = 7.8 Hz, 1H), 7.52 (m, 2H), 7.40 – 7.28 (m, 3H), 7.19 (m, 1H), 6.12 (p, *J* = 7.3 Hz, 1H), 3.14 – 3.00 (m, *J* = 7.4 Hz, 2H), 2.79 (s, 1H), 2.64 (m, 2H), 1.74 (d, *J* = 6.8 Hz, 3H). ^13^C NMR (126 MHz, Acetone, δ from solvent) δ 171.42, 169.30, 151.76, 140.76, 140.01, 138.07, 134.97, 132.12, 130.72, 130.42, 129.63, 128.52, 128.23, 127.15, 126.83, 126.52, 126.35, 124.37, 123.62, 79.90, 77.04, 76.70, 45.67, 36.17, 21.58. HRMS-ESI (*m/z*): [M + H]^+^ Theoretical for C_25_H_24_N_3_O_3_: 414.1819; Experimental: 414.1852.

#### (*R*)-2-(3-(2-(2-cyano-3-cyclopropylacryloyl)hydrazineyl)-3-oxopropyl)-*N*-(1-(naphthalen-1-yl)ethyl)benzamide (13).

^1^H NMR (500 MHz, Acetone-*d*_6_, δ from residual protio solvent) δ 8.32 (d, *J* = 8.6 Hz, 1H), 7.99 – 7.91 (m, 2H), 7.83 (d, *J* = 8.3 Hz, 1H), 7.71 – 7.68 (m, 2H), 7.63 – 7.58 (m, 1H), 7.57 – 7.46 (m, 2H), 7.39 (m, 1H), 7.32 (m, 2H), 7.21 (t, *J* = 7.1 Hz, 1H), 6.10 (p, *J* = 7.5 Hz, 1H), [1:2.5 E:Z isomer ratio; 4.51 (dd, *J* = 25.6, 7.6 Hz); 4.24 (dd, *J* = 54.2, 11.8 Hz, 1H)], 3.21 – 2.98 (m, 4H), 2.77 (s, 1H), 1.73 (d, *J* = 6.9 Hz, 3H), 1.18 – 1.02 (m, 1H), 0.70 – 0.56 (m, 2H), 0.56 – 0.41 (m, 2H). Many multiple peaks with close δ spacings were observed in the ^13^C NMR presumably due to the E:Z isomer mixture, these values are reported as observed. ^13^C NMR (126 MHz, Acetone, δ from solvent) δ 169.39, 169.21, 169.17, 169.15, 169.11, 169.06, 166.20, 166.09, 140.81, 140.80, 140.06, 140.05, 140.03, 139.99, 138.12, 138.09, 134.96, 132.10, 132.08, 131.02, 131.00, 130.47, 130.45, 129.68, 129.65, 128.53, 128.51, 128.50, 128.17, 128.14, 127.12, 127.11, 126.91, 126.51, 126.37, 126.32, 124.33, 124.31, 123.53, 123.49, 123.47, 115.74, 115.72, 114.81, 114.79, 64.23, 59.93, 45.69, 45.65, 45.62, 43.16, 43.13, 43.02, 42.97, 39.15, 39.11, 39.07, 30.30, 30.15, 29.99, 29.84, 29.69, 29.53, 29.38, 29.10, 28.56, 28.54, 21.64, 21.60, 12.09, 12.05, 12.02, 3.47, 3.23, 3.20, 2.90, 2.13, 2.10. HRMS-ESI (*m/z*): [M + H]^+^ Theoretical for C_29_H_29_N_4_O_3_: 481.2240; Experimental: 481.2289.

#### Preparation of noncovalent derivatives of GRL0617.

Compounds **14** and **15** were prepared analogously to the amide coupling of **3**. The amount of materials used were: 2-methylbenzoic acid (0.250 g, 1.80 mmol); 5-acetamido-2-methylbenzoic acid (0.348 g, 1.80 mmol); HBTU (0.853 g, 2.25 mmol); (*R*)-1-(naphthalen-1-yl)ethan-1-amine (0.306 g, 1.80 mmol) and DIPEA (0.392 mL, 2.25 mmol). Silica gel column purification was performed on **14** (3:1 Hexanes:EtOAc) and **15** (5% MeOH in DCM) to yield white solids **14** (0.463 g, 1.61 mmol, 89%); **15** (0.519 g, 1.50 mmol, 83%).

#### (*R*)-2-methyl-*N*-(1-(naphthalen-1-yl)ethyl)benzamide (14).

^1^H NMR (500 MHz, DMSO-*d*_6_, δ from residual protio solvent) δ 8.86 (d, *J* = 8.0 Hz, 1H), 8.25 (d, *J* = 8.4 Hz, 1H), 7.96 (d, *J* = 8.0 Hz, 1H), 7.85 (d, *J* = 8.1 Hz, 1H), 7.66 – 7.49 (m, 4H), 7.35 – 7.28 (m, 2H), 7.25 – 7.19 (m, 2H), 5.93 (p, *J* = 7.2 Hz, 1H), 2.30 (s, 3H), 1.59 (d, *J* = 6.9 Hz, 3H). ^13^C NMR (126 MHz, DMSO, δ from solvent)) δ 168.09, 140.25, 137.22, 135.01, 133.35, 130.40, 130.23, 129.07, 128.62, 127.18, 126.96, 126.08, 125.55, 125.43, 125.36, 123.17, 122.49, 44.26, 21.42, 19.21. HRMS-ESI (*m/z*): [M + H]^+^ Theoretical for C_20_H_20_NO: 290.1545; Experimental: 290.1594.

#### (*R*)-5-acetamido-2-methyl-*N*-(1-(naphthalen-1-yl)ethyl)benzamide (15).

^1^H NMR (500 MHz, DMSO-*d*_6_, δ from residual protio solvent) δ 9.91 (s, 1H), 8.85 (d, *J* = 8.1 Hz, 1H), 8.24 (d, *J* = 8.5 Hz, 1H), 7.96 (dd, *J* = 8.1, 1.6 Hz, 1H), 7.84 (d, *J* = 8.2 Hz, 1H), 7.64 – 7.45 (m, 7H), 7.12 (d, *J* = 8.3 Hz, 1H), 5.92 (p, *J* = 7.1 Hz, 1H), 3.29 (s, 1H), 2.69 (s with broadened couplings, 3H), 2.21 (s, 3H), 2.01 (d, *J* = 1.7 Hz, 3H), 1.57 (d, *J* = 6.9 Hz, 3H), 1.19 (s, 1H). ^13^C NMR (126 MHz, DMSO, δ from solvent) δ 168.12, 167.96, 140.20, 137.52, 136.78, 133.33, 130.40, 130.38, 129.10, 128.59, 127.18, 126.10, 125.55, 125.37, 123.15, 122.42, 119.51, 117.50, 44.15, 38.19, 23.84, 21.44, 18.51. HRMS-ESI (*m/z*): [M + H]^+^ Theoretical for C_22_H_23_N_2_O_2_: 369.1579; Experimental: 369.1555.

### Protein expression and purification.

PLpro from SARS-CoV-2 was produced using a previously described procedure with minor modifications,^43^ which we summarize here. First, the protein was expressed using *E. coli* BL21(DE3) cells that had been transformed with a pMCSG92 expression plasmid, which includes a T7 promoter and TEV protease-cleavable C-terminal 6xHis tag. Cells were plated on LB agar and cultivated in a shaking incubator (250 rpm) at 37°C in Lysogeny Broth medium (Lennox recipe) using 1 L per baffled 2.8 L Fernbach flask. Carbenicillin was used for antibiotic selection throughout. Bacterial growth was monitored by measuring the absorbance at 600 nm (OD_600_). Upon reaching an OD_600_ of ∼0.7, the incubator temperature was set to 18 °C and isopropyl β-D-1-thiogalactopyranoside (IPTG) was added to 0.2 mM. After approximately 18 hours, the culture was harvested by centrifugation at 6000×g for 30 minutes. After decanting off the supernatant, the pellets were stored at −80°C until needed for protein purification.

A cell pellet harvested from a 1 L culture was thawed and resuspended in 100 mL of lysis buffer containing 50 mM HEPES, 300 mM NaCl, 50 mM imidazole, 5% glycerol, and 1 mM TCEP at pH 7.4. Following resuspension, the cells were subjected to tip sonication on ice at 50% amplitude (2 seconds on and 10 seconds off) for a total sonication time of 5 minutes using a Branson 450D digital sonifier. After clarifying the lysate by 38,500xg centrifugation for 35 minutes at 4°C, the decanted supernatant was passed through 1.6- and 0.45-micron syringe filters sequentially and kept on ice while loading a 5-mL HisTrap HP column (Cytiva) at 2 mL/min. After washing the column with 10 column volumes (CV) of lysis buffer, partially purified PLpro was eluted using a linear gradient (20 CVs) of lysis buffer with 500 mM imidazole. Elution fractions (2 mL) were collected and PLpro was identified using SDS-PAGE on a 4–20% Mini-Protean TGX Stain-Free protein gel (Bio-Rad). Pooled fractions containing PLpro were dialyzed overnight at 6°C in 50 mM HEPES pH 7.4 with 150 mM NaCl, 5% glycerol, 20 mM imidazole, and 1 mM TCEP in the presence of His-tagged TEV protease (1 mg TEV protease:100 mg PLpro). After confirming His-tag cleavage by SDS-PAGE, the dialyzed protein solution was passed over a 5-mL HisTrap HP column to remove His-tagged impurities. The column flowthrough was collected, evaluated with SDS-PAGE, and concentrated with a 10-kDa molecular weight cutoff Amicon Ultra15 ultrafiltration membrane. Upon concentration, partially purified protein was applied at 0.5 mL/min to a Superdex 75 10/300 GL size-exclusion column (Cytiva) that had been equilibrated with 50 mM Tris HEPES pH 7.4 with 150 mM NaCl, 5% glycerol, and 1 mM TCEP. Fractions (0.5 mL) containing purified PLpro were collected, pooled, and concentrated for further use.

### PLpro inhibition assays.

The assays were performed in 40 μL total volume in black half area 96-well plates (Greiner PN 675076) at 25°C. The assay buffer contained 20 mM Tris-HCl pH 7.45, 0.1 mg/mL bovine serum albumin fraction V, and 2 mM reduced glutathione. The final DMSO concentration in all assays was 2.5% v/v. PLpro initial rates were measured using a previously established fluorogenic peptide substrate assay.^13, 19, 20^ The substrates Z-LRGG-AMC and Z-RLRGG-AMC were purchased from Bachem (PN 4027157 and 4027158), dissolved to 10 mM in DMSO and stored in aliquots at −20 °C. To determine Michaelis-Menten parameters, 20 μL enzyme solution was dispensed into wells (250 nM final concentration), and reactions were initiated by adding 20 μL substrate to 0–500 μM final concentration, in triplicate. Release of aminomethylcoumarin (AMC) was monitored by a Biotek Synergy H1 fluorescence plate reader every 50 s with an excitation wavelength of 345 nm and an emission wavelength of 445 nm, 6.25 mm read height, and gain = 60. After background subtraction of the average of no-enzyme negative controls, product formation was quantified using a 0.02–5 μM calibration curve of AMC (Sigma PN 257370). Initial rates were determined for time points in the initial linear range by linear regression in Excel, and GraphPad Prism 9 was used to perform nonlinear regression of the Michaelis-Menten equation to the initial rate vs. substrate concentration data to yield *K*_M_ and *V*_max_.

Inhibitors were characterized by dispensing 10 μL enzyme solution into wells (115 nM final concentration), followed by 10 μL inhibitor solution at 4x desired final concentrations in 5% v/v DMSO in at least duplicate, centrifuging briefly, and incubating for 30 min. Reactions were initiated by adding 20 μL substrate to 100 μM final concentration. Initial rates were determined as described above and % residual activities were determined by normalizing to the average of no inhibitor controls (100% activity). Thirty-minute IC_50_ values were determined by nonlinear regression to the [Inhibitor] vs. normalized response – Variable slope equation using GraphPad Prism 9.

Time-dependent inhibition assays were performed as described above, except that preincubation times were varied by adding the inhibitor to the enzyme at specific time points. For each inhibitor concentration, initial rates were normalized such that 0 preincubation time is 100% and plotted against preincubation time. A nonlinear regression to a one phase decay model was performed to determine the rate constants *k*_obs_ for each concentration and their 95% confidence intervals. These rate constants were then plotted against inhibitor concentration, and the data in the initial linear region was fit to determine the slope, which is *k*_inact_/*K*_I_. All regressions were performed with GraphPad Prism 9.

### Mass spectrometry to assess covalent adduct formation.

A Waters Synapt HDMS QTOF mass spectrometer was used to measure the intact protein mass of PLpro with and without preincubation with inhibitors to detect covalent adduct formation. To prepare the samples, 2 μL of 20 mM inhibitor stocks in DMSO were added to 100 μL PLpro at 1 mg/mL concentration and incubated 1 h at room temperature. Previously described protocols for ultrafiltration and denaturing direct infusion^44^ were implemented as follows. Samples were processed by ultrafiltration with a Vivaspin 500 10 kDa PES membrane by diluting the sample to 0.5 mL with 10 mM LC-MS grade ammonium acetate and reducing volume to 50 μL twice, followed by the same procedure with 2.5 mM ammonium acetate. Protein concentrations were estimated by A280 with a NanoDrop 2000, and samples were diluted to 2 mg/mL in 2.5 mM ammonium acetate, and then 10 μL were further diluted into 90 μL 50:50 acetonitrile:water with 0.1% formic acid. Sample was introduced into the electrospray ionization source by syringe pump at a flow rate of 10 μL/min and MS1 spectra were collected for m/z 400–1500, 5 s/scan, for 1 min. The protein monoisotopic mass was determined from the averaged spectra using mMass 5.5.^45^

### Inhibition of PLpro deubiquitinase and de-ISG15ylase activities and deubiquitinase selectivity.

Candidate inhibitors were assayed by LifeSensors, Inc. (Malvern, PA) in quadruplicate for inhibition of SARS-CoV-2 PLpro with Ub-rhodamine and ISG15-CHOP2 and with human deubiquitinase (DUB) enzymes, including USP30, 15, 8, 7, 4, and 2C as well as UCHL1 with Ub-rhodamine, except for USP7, which was tested with Ub-CHOP2. The CHOP assay^46^ uses a quenched enzyme platform to quantify the DUB inhibition activity of the compounds. In this assay, a reporter enzyme is fused to the C-terminus of ubiquitin. The reporter is silent when fused to ubiquitin but becomes fluorescent upon cleavage from the C-terminus by a DUB. Thus, measurement of the reporter activity is a direct measure of DUB activity. See Extended Data File 2 for report, which includes additional method details.

### PLpro expression, purification, and crystallization.

Wild-type PLpro from SARS-CoV-2 was expressed in BL21(DE3) *E. coli* cells transformed with the pMCSG53 expression plasmid with a T7 promoter and a TEV-cleavable, N-terminal 6xHis-tagged PLpro. *E. coli* cells were grown in LB media containing 50 µg/mL ampicillin at 37 °C in a shaking incubator (200 rpm) until the optical density (OD_600_) of the culture was 0.6. The culture was then induced with 0.5 mM IPTG (GoldBio, USA) and grown for 16 hours at 18 °C. The culture was centrifuged for 15 min at 3000x g and the cells were obtained as pellets. *E. coli* pellets were resuspended in lysis buffer (50 mM HEPES pH 7.2, 150 mM NaCl, 5% glycerol, 20 mM imidazole, 10 mM 2-mercaptoethanol) and subjected to sonication for cell lysis. The soluble fraction of the whole cell lysate was separated by centrifugation at 20442×g for 80 minutes and was loaded onto a Ni-NTA Agarose (Qiagen, USA) gravity column pre-equilibrated with lysis buffer. The column was washed with 25 column volumes of wash buffer (50 mM HEPES pH 7.2, 150 mM NaCl, 5% glycerol, 50 mM imidazole, 10 mM 2-mercaptoethanol) and eluted in fractions with elution buffer (50 mM HEPES pH 7.2, 150 mM NaCl, 5% glycerol, 500 mM imidazole, 10 mM 2-mercaptoethanol). Fractions containing PLpro protein as determined by SDS-PAGE were combined and dialyzed overnight in dialysis buffer (50 mM HEPES pH 7.2, 150 mM NaCl, 5% glycerol, 10 mM 2-mercaptoethanol). Dialyzed PLpro was mixed with 6xHis-tagged TEV protease in 25:1 ratio, incubated overnight at 4 °C and was passed through Ni-NTA Agarose (Qiagen, USA) gravity column pre-equilibrated with dialysis buffer (50 mM HEPES pH 7.2, 150 mM NaCl, 5% glycerol, 10 mM 2-mercaptoethanol) to remove 6xHis-tagged impurities and TEV protease. Tagless PLpro obtained as the flowthrough was flash frozen and stored at −80 °C. All extraction and purification steps were performed at 4 °C. Reaction of tag-less PLpro in 20 mM Tris HCl pH 8.0 and 5 mM NaCl with a 10-fold molar excess of compound **7** was performed at 37 °C for 20 minutes. The PLpro-compound **7** complex in a solution containing 20 mM Tris HCl, 100 mM NaCl and 10 mM DTT was then used for crystallization at a concentration of 8 mg/ml. Initial crystal hits were obtained by screening around 900 crystallization conditions by the sitting drop method. Diffraction-quality crystals were obtained from a well solution containing PEG-3350, CaCl_2_, CdCl_2_ and CoCl_3_.

### Data collection and structure determination.

The diffraction data were collected at 100 K at the BL12–2 beamline of the Stanford Synchrotron Radiation Light Source using Pilatus 6M detectors. Crystals for the complex were cryo-cooled using the well solution supplemented with 20% ethylene glycol. Diffraction data from two crystals were collected with 360 degrees of data per crystal and 0.2 degrees oscillation per image. For each crystal, diffraction data were merged and processed with the XDS suite of programs.^47^ The structures were solved by molecular replacement with AMoRE^48^ using the coordinates of SARS-CoV-2 PLpro complexed with the tetrapeptide-based inhibitor VIR251 (PDB 6WX4^9^) as the search model. Iterative rounds of model building and refinement were performed with the programs COOT^49^ and REFMAC.^50^ The details of data collection and refinement for the higher resolution data (3.10 Å) are presented in Extended Data Table S2.

### SARS-CoV-2 antiviral assays.

Initial screening to measure cytopathic effect (CPE) protection for the 50% efficacy concentration (EC_50_) and cytotoxicity (CC_50_) was performed using an assay based on African green monkey kidney epithelial (Vero E6) cells in 384-well plates.^51^ Each plate can evaluate 5 compounds in duplicate at 7 concentrations to measure an EC_50_ and CC_50_. Each plate included three controls: cells alone (uninfected control), cells with SARS-CoV-2 (infected control) for plate normalization, and remdesivir as a drug control. Cell viability was measured using the CellTiter-Glo Luminescent Cell Viability Assay (Promega). In brief, Vero E6 TMPRSS ACE2 cells were grown to ∼90% confluency in 384-well plates and treated for 1 hr with compounds selected in Aim 2. Cells were infected at an MOI = 0.1 of SARS-CoV-2 isolate USA-WA1/2020.^52^ After 48 h, the SARS-CoV-2-mediated CPE or cytotoxicity was assessed by measurement of live cells using CellTiter-Glo. The selectivity index at 50% (SI_50_) was then calculated from the EC_50_ and CC_50_ values. To ensure robust and reproducible signals, each 384-well plate was evaluated for its Z-score, signal to noise, signal to background, and coefficient of variation. This assay has been validated for use in high-throughput format for single-dose screening and is sensitive and robust, with Z values > 0.5, signal to background > 20, and signal to noise > 3.3.

### Metabolic stability.

Intrinsic clearance in human, Sprague-Dawley rat, and CD-1 mouse liver microsomes and S9 fractions were measured^53^ in duplicate for compounds **7**, **9**, and **14** by Eurofins Panlabs (St. Charles, MO, USA). Imipramine, propranolol, terfenadine, and verapamil were used as reference compounds at a test concentration of 0.1 mM. In each experiment and if applicable, the respective reference compounds were tested concurrently with the test compounds, and the data were compared with historical values determined at Eurofins. The experiment was accepted in accordance with Eurofins validation Standard Operating Procedure. Metabolic stability, expressed as percent of the parent compound remaining, was calculated by comparing the peak area of the compound at the time point relative to that at time t_0_. The concentration of each compound was 1 mM and the incubation time ranged from 0 to 60 min. The half-life (T_1/2_) was estimated from the slope of the initial linear range of the logarithmic curve of compound remaining (%) versus time, assuming first-order kinetics. The apparent intrinsic clearance (CL_int_, μL/min/mg) was then calculated according to the following formula:

$${\text{CL}}_{\text{int}}=\frac{0.693}{{\text{T}}_{1/2}\left(mg\;protein/\text{μL}\right)}$$

## Supplementary Material

Supplement 1

Supplement 2

Supplement 3

## Figures and Tables

**Figure 1:**
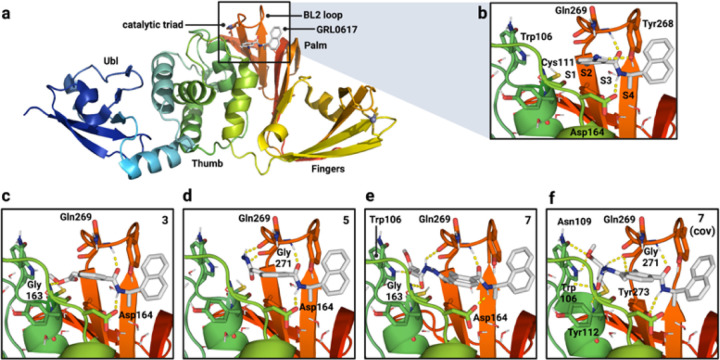
a, Structure and domains of PLpro from SARS-CoV-2 (PDB entry 7JIR12). Selected features are labeled. b, Interactions between PLpro and the noncovalent inhibitor GRL0617. Docked poses of: c, compound 3; d, compound 5; and compound 7 docked e, noncovalently and f, covalently. Polar hydrogens have been added in b-f. Docked poses for additional inhibitor candidates are depicted in Extended Data Fig. S2. Ligand carbons are shown in gray and predicted protein-ligand interactions are shown as dashed yellow lines.

**Figure 2:**
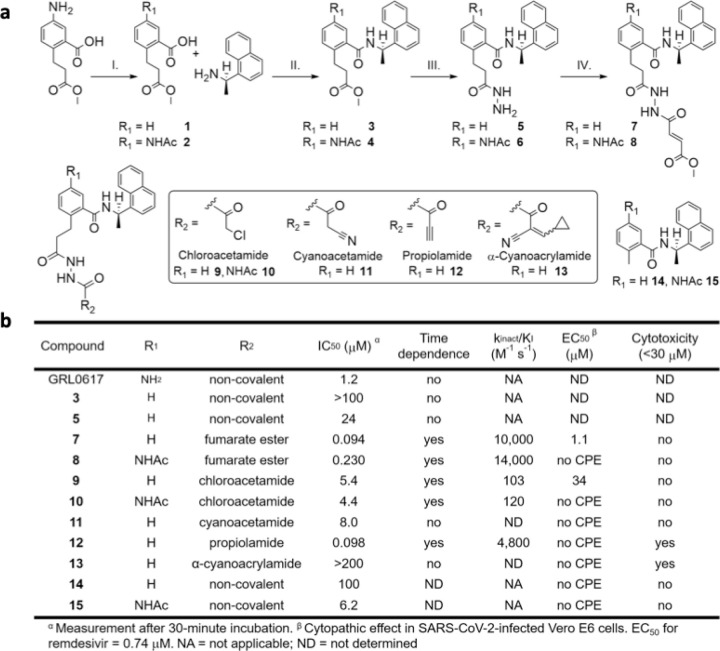
a, Synthesis of compounds 2–13. Reaction conditions: (I.) Ac2O, AcOH, DCM; (II.) HATU, DIPEA, DCM; (III.) N2H4•H2O, EtOH; (IV.) methyl (E)-4-chloro-4-oxobut-2-enoate, DIPEA, DCM for 7, and K2CO3, DMF for 8. Compounds 9–13 were prepared with the corresponding acid chlorides under conditions described for step IV. Compounds 14 and 15 were prepared analogously to step II with 2-methylbenzoic acid and 5-acetamido-2-methylbenzoic acid, respectively. b, PLpro inhibition and SARS-CoV-2 antiviral activity.

**Figure 3:**
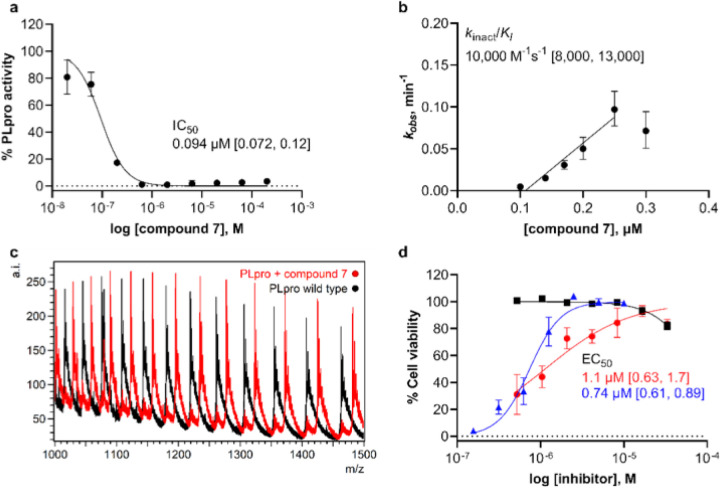
Characterization of designed covalent PLpro inhibitor, compound 7. a, Fluorogenic peptide activity assay after 30-min preincubation with fumarate methyl ester 7. Data points are the average of n = 2 independent samples ± range and are representative of n = 3 independent experiments. IC50 is the concentration at which 50% inhibition was observed, and bracketed values are the 95% confidence interval. Curve is the nonlinear regression to the normalized inhibitor dose response equation. b, Time-dependent characterization with a fluorogenic peptide assay. Data points are kobs values determined by fitting the exponential decay equation to initial rates determined at various inhibitor concentrations and preincubation times, normalized to no preincubation. kobs values were determined from n = 2 independent experiments with n = 2 independent samples each ± 95% confidence interval of the nonlinear regression. Line represents the linear regression yielding as its slope the second-order rate constant (kinact/KI). c, Intact protein ESI-MS spectra of PLpro (black) and PLpro incubated with 7 (red); a.i., arbitrary intensity; m/z, mass-to-charge ratio. d, Cell viability in Vero E6 cells for uninfected cells pretreated with 7 (black squares), SARS-CoV-2-infected cells pretreated with 7 (red circles) and with remdesivir drug control (blue triangles). Data points are the average of n = 2 independent samples ± range and are representative of n = 2 independent experiments. EC50 is the concentration at which 50% effect was observed and bracketed values are the 95% confidence interval. Curves are nonlinear regressions to the normalized dose response equation.

**Figure 4:**
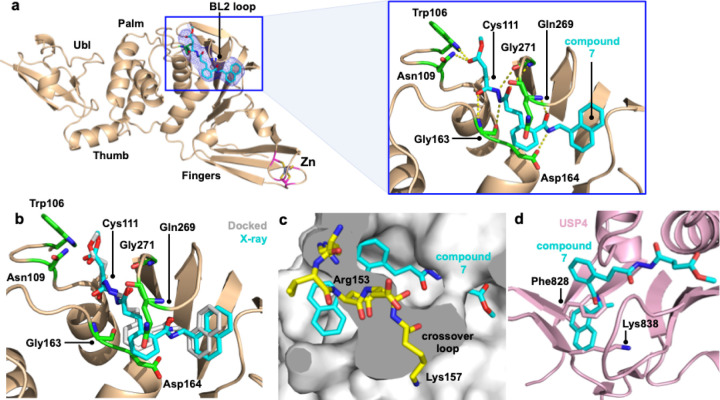
Crystal structure of SARS-CoV-2 PLpro in complex with inhibitor 7. a, Overall structure and interactions between the active site residues and 7 (cyan sticks). The electron density for 7 is shown in blue mesh (Fo - Fc omit map contoured at 1.5 σ). b, Superposition of the covalently docked model of 7 (grey sticks) and the co-crystal structure of PLpro and 7 (cyan sticks). c, Structural basis for selectivity toward PLpro. Superposition of 7 bound to PLpro onto human deubiquitinase UCHL122 (PDB entry 3KW5). The crossover loop of UCHL1, 153-RVDDK-157, covers the narrow groove and blocks the naphthylmethylamine core of 7 from binding. The crossover loop is longer and, in some cases, more disordered in UCHL3 and UCHL5 (see for example ref 23). d, Superposition of 7 bound to PLpro onto human USP424 (PDB entry 2Y6E). Severe steric clashes are present between the naphthyl ring of 7 and Phe828 and Lys838 of USP4 (light pink sticks), both of which are conserved in 80% of human USPs.
