# Supplementary material for: Potent and Selective Covalent Inhibitors of the Papain-like Protease from SARS-CoV-2
Source: Res Sq. 2021 Oct 8:rs.3.rs-906621. Preprint. [Version 1] doi: 10.21203/rs.3.rs-906621/v1 (PMC8509099; doi:10.21203/rs.3.rs-906621/v1)
Supplement: Supplementary file 1 [file b4653e841e0af6bc78a7000a.docx]

**Extended Data**

**Extended Data Fig. S1**. Superposition of 50 ensemble models and docking box used for docking. Conserved water molecules are shown in stick representation.

**Extended Data Fig. S2**. Docked poses of compounds **8**-**13**. Left: noncovalent, right: covalent.

**Extended Data Fig. S3**. Summary of docking results for experimentally tested compounds. Docking scores are in kcal/mol and MCS-RMSDs are in Å. Additional docking data for all candidate inhibitors are provided in **Extended Data File 1**. 

**Extended Data Fig. S4**. Michaelis-Menten kinetics data for Z-RLRGG-AMC and Z-LRGG-AMC substrates. Data points represent the average initial rate for *n* = 2 independent experiments performed with *n* = 3 replicates, ± s.d. 

**Extended Data Fig. S5.** Fluorogenic peptide activity assay after 30 min preincubation with inhibitor candidates. Data points are the average of *n* = 2 independent samples ± range and are representative of *n* ≥ 1 independent experiments. IC_50_ is the concentration at which 50% inhibition was observed, and bracketed values are the 95% confidence interval. Curve is the nonlinear regression to the normalized inhibitor dose response equation.

**Extended Data Fig. S6.** Time-dependent characterization with fluorogenic peptide assay. **a**, **c**, **e**, **g**, and **i** are initial rates determined at various inhibitor concentrations (concentrations in μM, inhibitor in x-axis of right panels) and preincubation times, normalized to no preincubation. Data points are from *n* ≥ 2 independent experiments with n = 2 independent samples each, ± range. Curves are nonlinear regressions of the exponential decay equation to the data. **b**, **d**, **f**, **h**, and **j**, Data points are the *k*_*obs*_ values determined from **a**, **c**, **e**, **g**, and **i** ± 95% confidence interval of the nonlinear regression. Line represents the linear regression yielding as its slope the second-order rate constant (*k*_*inact*_*/K*_*I*_), with bracketed values representing 95% confidence interval of the slope.

**Extended Data Fig. S7**. Time-dependent characterization with fluorogenic peptide assay. **a**, compound **11**; **b**, compound **13**. Data points are from an experiment with n = 2 independent samples, ± range. 

**Extended Data Fig. S8**. Intact protein ESI-MS spectra of PLpro (black) and PLpro incubated with compounds indicated in figure legend (red). a.i., arbitrary intensity; m/z, mass-to-charge ratio. Spectral peaks used for protein monoisotopic mass deconvolution are annotated with their monoisotopic masses and, in parentheses, charge states.

**Extended Data Fig. S9**. Cell viability in Vero E6 cells for uninfected cells pretreated with indicated compound (black) and SARS-CoV-2-infected cells pretreated with indicated compound (red). Data points are the average of *n* = 2 independent samples ± range and are representative of *n* ≥ 1 independent experiments. EC_50_ is the concentration at which 50% effect was observed, CC_50 _is the concentration at which 50% cytotoxicity was observed, and bracketed values are the 95% confidence intervals. Curves are nonlinear regression to the normalized dose response equation.

| **Extended Data Table S1.** PLpro intact protein ESI-MS analysis. |  |  |  |
| --- | --- | --- | --- |
| **Compound** | **Deconvoluted monoisotopic PLpro+compound mass** | **Measured** **adduct** **mass** | **Predicted** **adduct** **mass** |
| - (wild-type PLpro) | 36535 (FWHM 16) | - | - |
| **7** | 37009 (FWHM 16) | 474 | 473 |
| **8** | 37066 (FWHM 16) | 531 | 530 |
| **9** | 36936 (FWHM 15) | 401 | 401 |
| **10** | 36995 (FWHM 15) | 460 | 458 |
| FWHM, full-width half maximum |  |  |  |

**Extended Data Table S2**. Summary of X-ray crystallography data collection and refinement

|  | PLpro-compound **7** |
| --- | --- |
| **Crystallographic parameters** Space group Unit-cell dimensions | I4_1_22 112.33 Å, 112.33 Å, 217.33 Å 90.0˚, 90.0˚, 90.0˚ |
| Data collection statistics Resolution limits (Å) No. of observed reflections No. of unique reflections Completeness      overall/outer shell CC1/2 (overall/outer shell) R_sym_^a^ (%)      overall/outer shell & os I/σ | 39.1 – 3.10 333876 13010 99.8/99.8 99.8/75.2 44.5/720.0 & 1.3 |
| Refinement statistics Resolution limits (Å) Number of reflections/% (│F│ > 2σ│F│) Reflections used for R_free_ R_factor_^b^ (%) R_free_ (%) Model contents/average B (Å^2^)    Protein atoms    Ligand    Ions    Water molecules RMS deviations    Bond length (Å)    Bond angle (°) Ramachandran (analyzed/outliers) | 39.1-3.10 12359/99.8 651 19.1 25.3 2468/97.4 35/81.7 8/101.9 42/71.6 0.008 1.79 311/5 |

**Extended Data Table S3**. Metabolic stability in liver microsomes

|  | human |  | rat |  | mouse |  |
| --- | --- | --- | --- | --- | --- | --- |
|  | half-life (min)^a^ | Cl_int_ (μL/min/mg) | half-life (min)^a^ | Cl_int_ (μL/min/mg) | half-life (min)^a^ | Cl_int_ (μL/min/mg) |
| **7** | 50 | 138.8 | 46 | 151.2 | 13 | 539.7 |
| **9** | 7 | 974.9 | 13 | 514.7 | 5 | 1333.3 |
| **14** | 41 | 171.0 | 21 | 329.7 | 16 | 434.6 |
| imipramine | >60 | <115.5 | 5 | 1420.5 | 16 | 435.5 |
| propranolol | >60 | <115.5 | 3 | 2387.8 | 20 | 373.2 |
| terfenadine | 11 | 615.1 | 7 | 1053.6 | 8 | 851.4 |
| verapamil | 38 | 183.0 | 35 | 195.7 | 18 | 374.8 |

^a^ mean of two replicates

**Extended Data Table S4**. Metabolic stability in liver S9 fractions

|  | human |  | rat |  | mouse |  |
| --- | --- | --- | --- | --- | --- | --- |
|  | half-life (min)^a^ | Cl_int_ (μL/min/mg) | half-life (min)^a^ | Cl_int_ (μL/min/mg) | half-life (min)^a^ | Cl_int_ (μL/min/mg) |
| **7** | 60 | 38.9 | 31 | 75.5 | 9 | 266.4 |
| **9** | 3 | 689.4 | 16 | 140.4 | 4 | 544.0 |
| **14** | >60 | <38.5 | 17 | 137.5 | 18 | 128.4 |
| imipramine | >60 | <38.5 | 3 | 886.4 | 15 | 153.3 |
| propranolol | >60 | <38.5 | 3 | 765.7 | 6 | 356.6 |
| terfenadine | 16.0 | 144.5 | 8 | 290.8 | 14 | 160.1 |
| verapamil | >60 | <38.5 | 31 | 76.1 | 18 | 127.0 |

^a^ mean of two replicates

**Extended Data Figure S10**. MetaSite site-of-metabolism prediction for compound **7**. The shade of the red circles indicates relative liability (darker = greater liability).
