# Supplementary material for: Potent and Selective Covalent Inhibitors of the Papain-like Protease from SARS-CoV-2
Source: Res Sq. 2021 Oct 8:rs.3.rs-906621. Preprint. [Version 1] doi: 10.21203/rs.3.rs-906621/v1 (PMC8509099; doi:10.21203/rs.3.rs-906621/v1)
Supplement: Supplementary file 1 [file 8eba726a1313e67b8d7b0302.pdf]

LifeSensors www.lifesensors.com

Oak Ridge National Laboratories

Lot # 04/2021
Issue Date: 26-May-21
Revision Date:

**Assay results:**	**BCS 065**	**BCS 067**	**BCS 071**
**PLpro (Ub-Rhodamine) IC_{50} (M)**	>3.0E-05	1.9E-06	7.6E-08
**PLpro (ISG15-CHOP2) IC_{50} (M)**	>3.0E-05	2.0E-07	3.7E-08
**USP30 IC_{50} (M)**	>3.0E-05	>3.0E-05	>3.0E-05
**USP15 IC_{50} (M)**	>3.0E-05	>3.0E-05	>3.0E-05

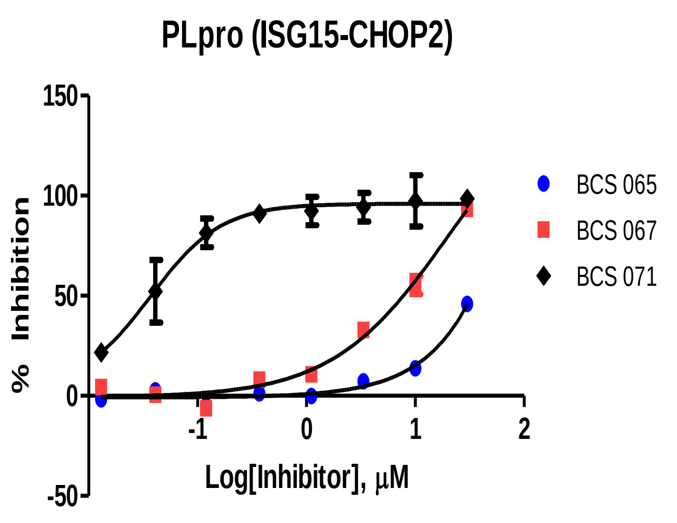

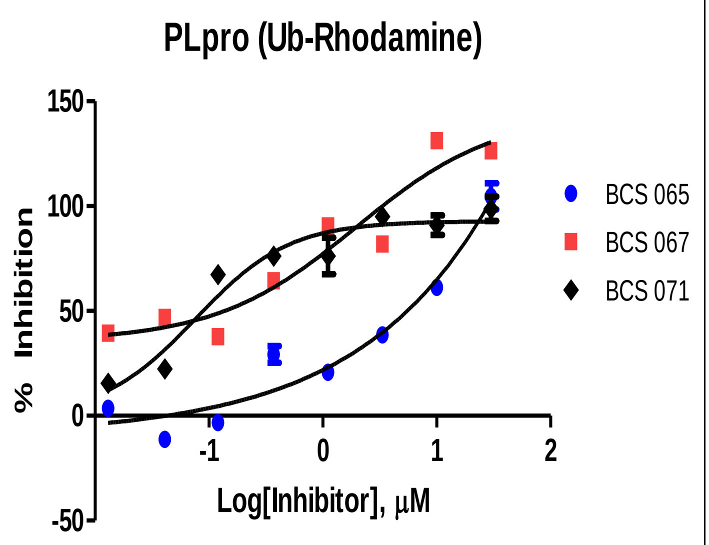

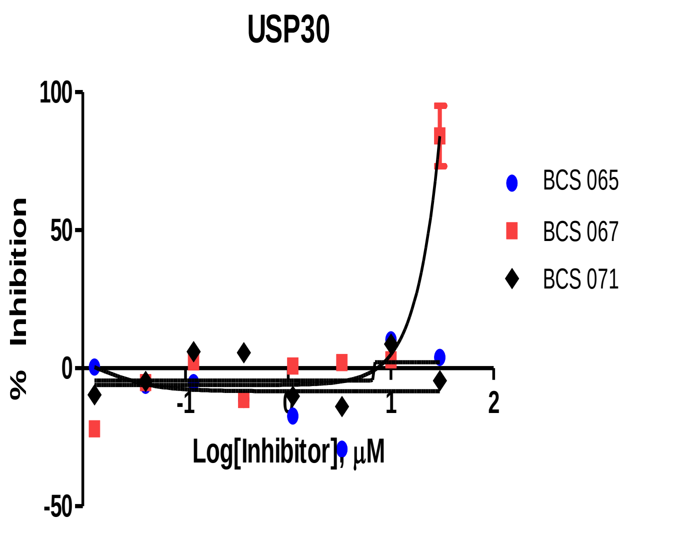

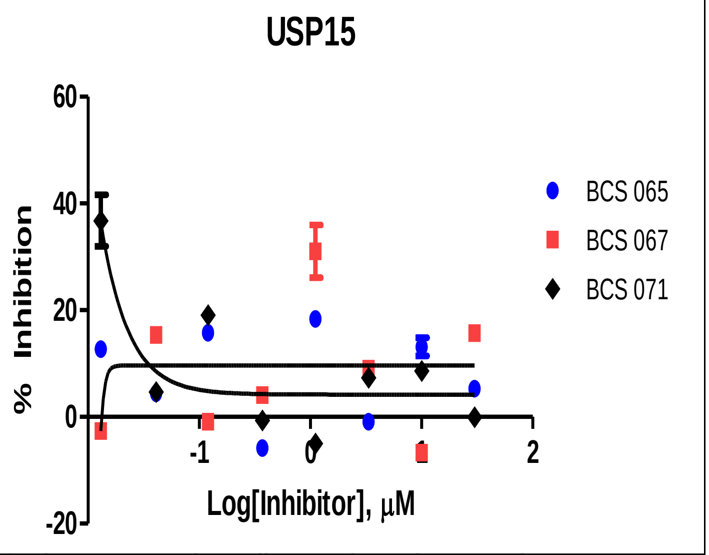

All products are for research use only • not intended for human or animal diagnostic or therapeutic uses
Copyright © 2016 LifeSensors, Inc. All Rights Reserved

Assay results:	BCS 065	BCS 067	BCS 071
USP8 IC_{50} (M)	>3.0E-05	>3.0E-05	>3.0E-05
USP7 IC_{50} (M)	>3.0E-05	>3.0E-05	>3.0E-05
USP4 IC_{50} (M)	>3.0E-05	>3.0E-05	>3.0E-05
USP2 IC_{50} (M)	>3.0E-05	>3.0E-05	>3.0E-05
UCHL1 IC_{50} (M)	>3.0E-05	>3.0E-05	>3.0E-05

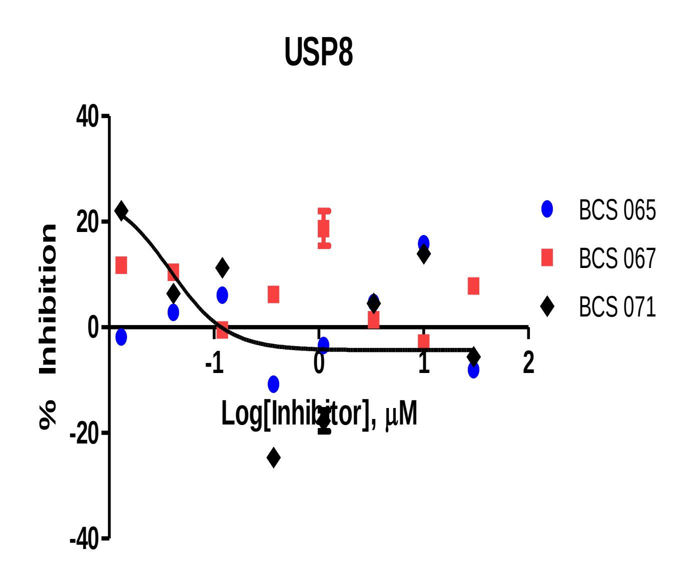

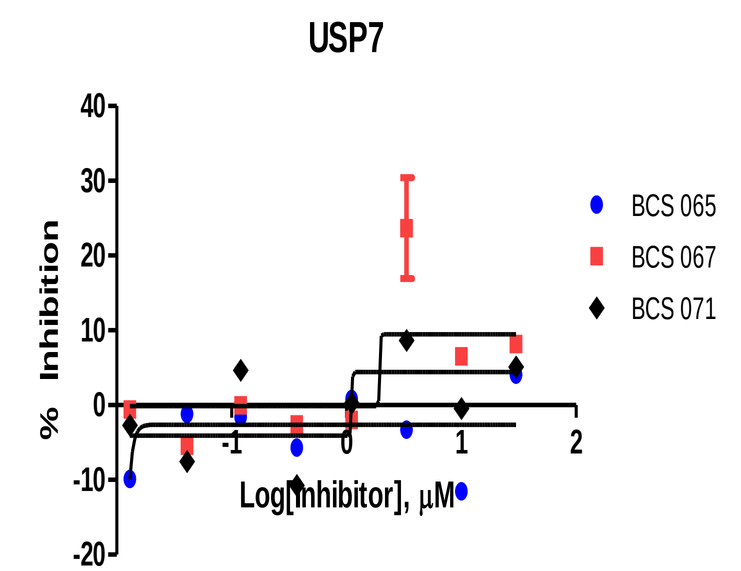

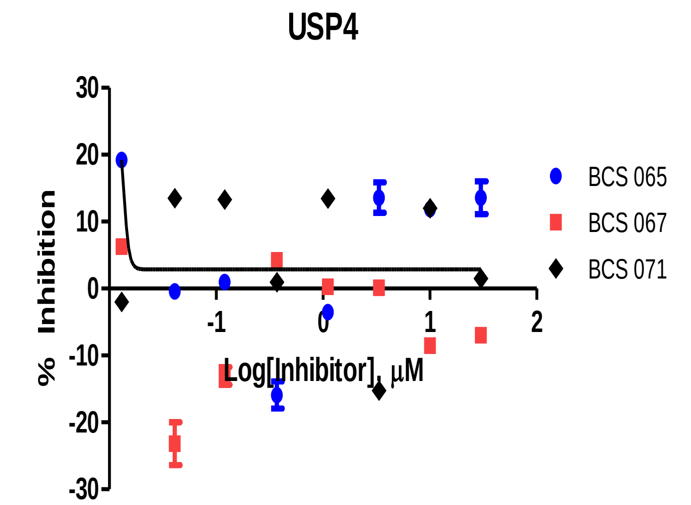

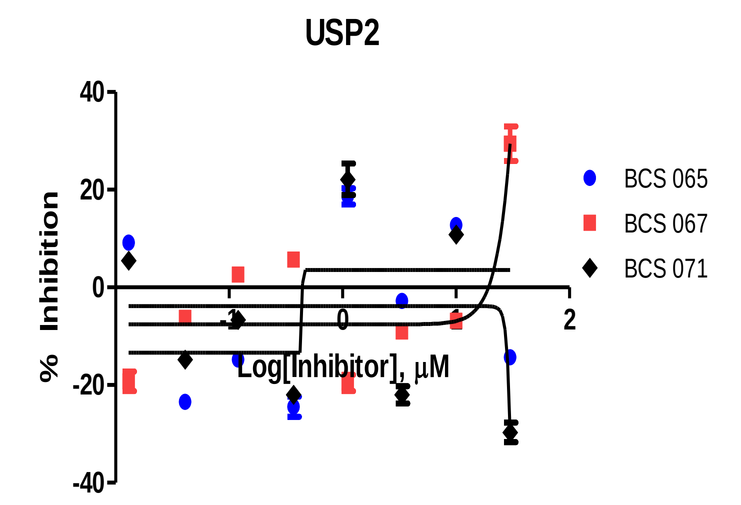

All products are for research use only • not intended for human or animal diagnostic or therapeutic uses
Copyright © 2016 LifeSensors, Inc. All Rights Reserved

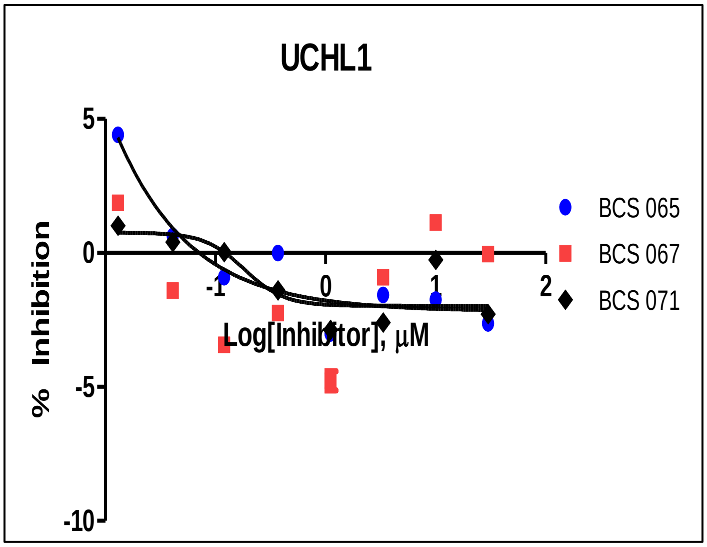

Summary of IC50s with Selectivity Index

	BCS 065			BCS 067			BCS 071		
DUB	IC50, µM	Hill Slope	Selectivity Index	IC50, µM	Hill Slope	Selectivity Index	IC50, µM	Hill Slope	Selectivity Index
PLpro (Ub-Rhodamine)	>30	0.376	1	1.96	0.705	1	0.076	1.06	1
PLpro (ISG15-EKL)	>30	0.969	1	20.2	0.817	1	0.037	1.34	1
USP30	>30	N/A	1	>30	N/A	15.3	>30	N/A	394.7
USP15	>30	N/A	1	>30	N/A	15.3	>30	N/A	394.7
USP8	>30	N/A	1	>30	N/A	15.3	>30	N/A	394.7
USP7	>30	N/A	1	>30	N/A	15.3	>30	N/A	394.7
USP4	>30	N/A	1	>30	N/A	15.3	>30	N/A	394.7
USP2	>30	N/A	1	>30	N/A	15.3	>30	N/A	394.7
UCHL1	>30	N/A	1	>30	N/A	15.3	>30	N/A	394.7

All products are for research use only • not intended for human or animal diagnostic or therapeutic uses
Copyright © 2016 LifeSensors, Inc. All Rights Reserved

CONTACT: LifeSensors, Inc. | 271 Great Valley Parkway | Malvern, PA 19355 | 610.644.8845 | www.lifesensors.com

Experimental Design and Enzyme Panels

Lifesensors performed all the assays in quadruplicate, in 384 well plates. Positive control (PR619) and negative control (without the inhibitor) were performed. DUBs at previously optimized concentration were used with previously optimized suitable DUB substrates to evaluate the inhibition activity. Briefly, the received compound in DMSO was thawed before use and simultaneously aliquoted to protect from deterioration from freeze-thaw cycles. Compound were diluted at desired fold to perform a dose response curve in DMSO. DMSO control is used as 0% inhibition in presence of DUB and DMSO control without DUB is considered as 100% inhibition control to calculate IC_{50}s. Dose response – inhibition curves were plotted on prism with log transformed concentration on X-axis with percentage inhibition (30 min time point) on Y-axis using Log[inhibitor] Vs response-variable slope. Data represented for each of the enzyme with both PR619 and Test compound in a same graph along with calculated IC50s with hill slope values. Hill slope can be considered as an interaction coefficient that reflects binding of ligands to proteins. **Hill Slope of 1** indicates independent binding, a value greater than **1** indicates positive cooperativity in which binding of **one** ligand facilitates binding of subsequent ligands at other sites; a value less than **1** indicates negative cooperativity. Selectivity index is represented as fold change in selectivity for PLpro compared to DUB inhibition activity of other DUBs in the selectivity panel.

Basic Panel*

Enzyme name	DUB substrate
PLpro, 50nM	Ub-Rhodamine110
PLpro, 10nM	ISG15-CHOP2
USP30, 10 nM	Ub-Rhodamine 110
USP15, 1 nM	Ub-Rhodamine 110
USP8, 10 nM	Ub-Rhodamine 110
USP7, 5 nM	Ub-CHOP2
USP4, 10nM	Ub-Rhodamine 110
USP2c, 10 nM	Ub-Rhodamine 110
UCHL1, 10nM	Ub-Rhodamine 110

Summary

Test compounds BCS-065, BCS-067, and BCS-071 demonstrated selective inhibition of PLpro, compared to other DUBs in the selective panel. BCS-071 performed better in inhibition of PLpro in both Ub-Rho110 and ISG15-CHOP2 assay with IC_{50}s of 76nM and 37nM, respectively. BCS-067 demonstrated IC_{50} at 1.96µM for Ub-Rhodamine and 20.2µM for ISG15-CHOP2. No appreciable IC50 was obtained for BCS-065 compound for inhibition of PLpro. No DUBs across the selectivity panel were inhibited by these test compounds. Comparison of the results between PLpro substrates Ub-Rhodamine and ISG15-CHOP2 show the same rank order in potency among the compounds.

All products are for research use only • not intended for human or animal diagnostic or therapeutic uses
Copyright © 2016 LifeSensors, Inc. All Rights Reserved

LifeSensors www.lifesensors.com

Supplementary Info:

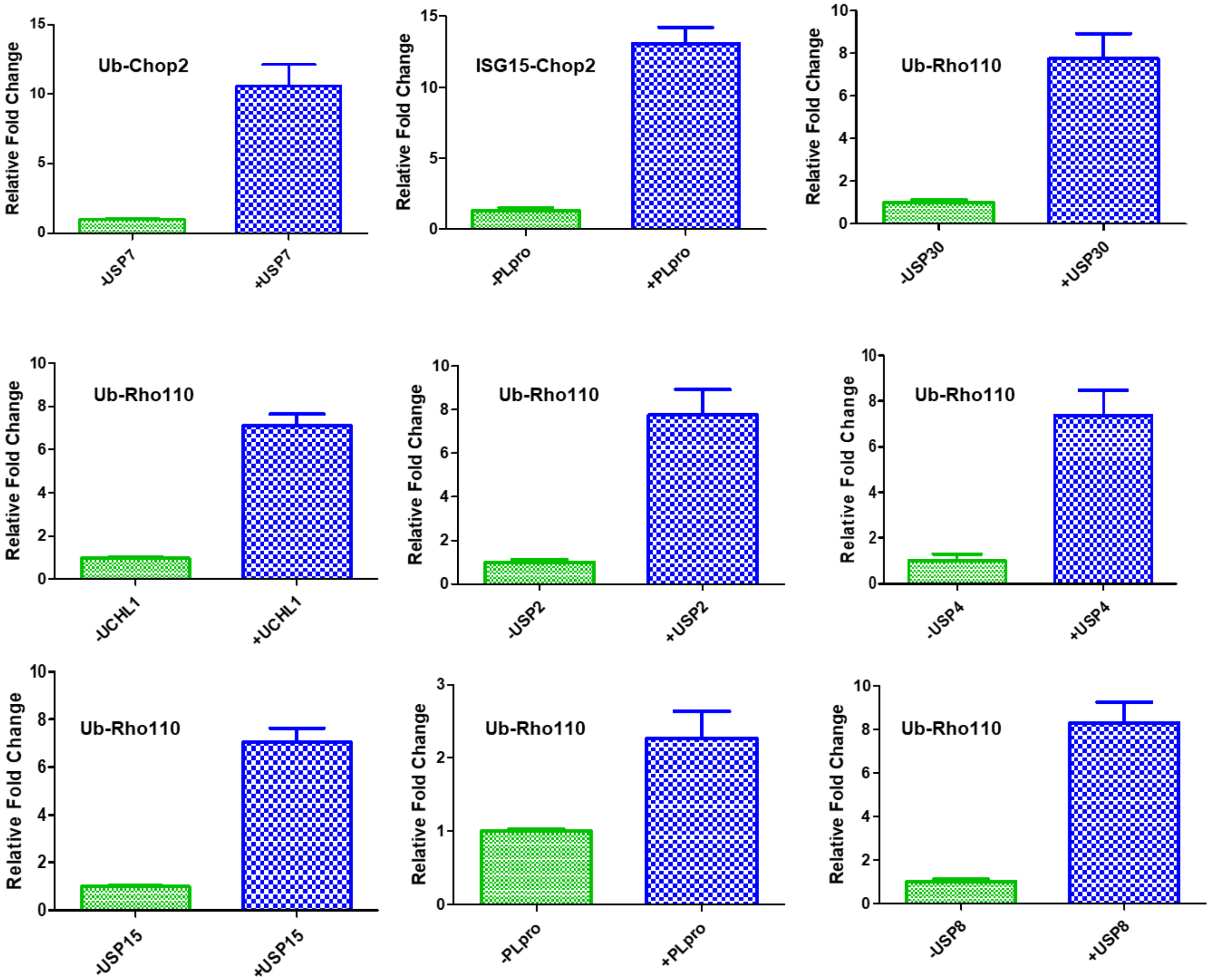

SI. Fig 1: Enzyme activity and substrate compatibility demonstrated as relative fold chance. -DUB represents (100% inhibition) the relative signal in presence of DMSO and substrate. +DUB represents (0% Inhibition), the relative fold signal enhancement in presence of DMSO and substrate.

All products are for research use only • not intended for human or animal diagnostic or therapeutic uses
Copyright © 2016 LifeSensors, Inc. All Rights Reserved

CONTACT: LifeSensors, Inc. | 271 Great Valley Parkway | Malvern, PA 19355 | 610.644.8845 | www.lifesensors.com

LifeSensors www.lifesensors.com

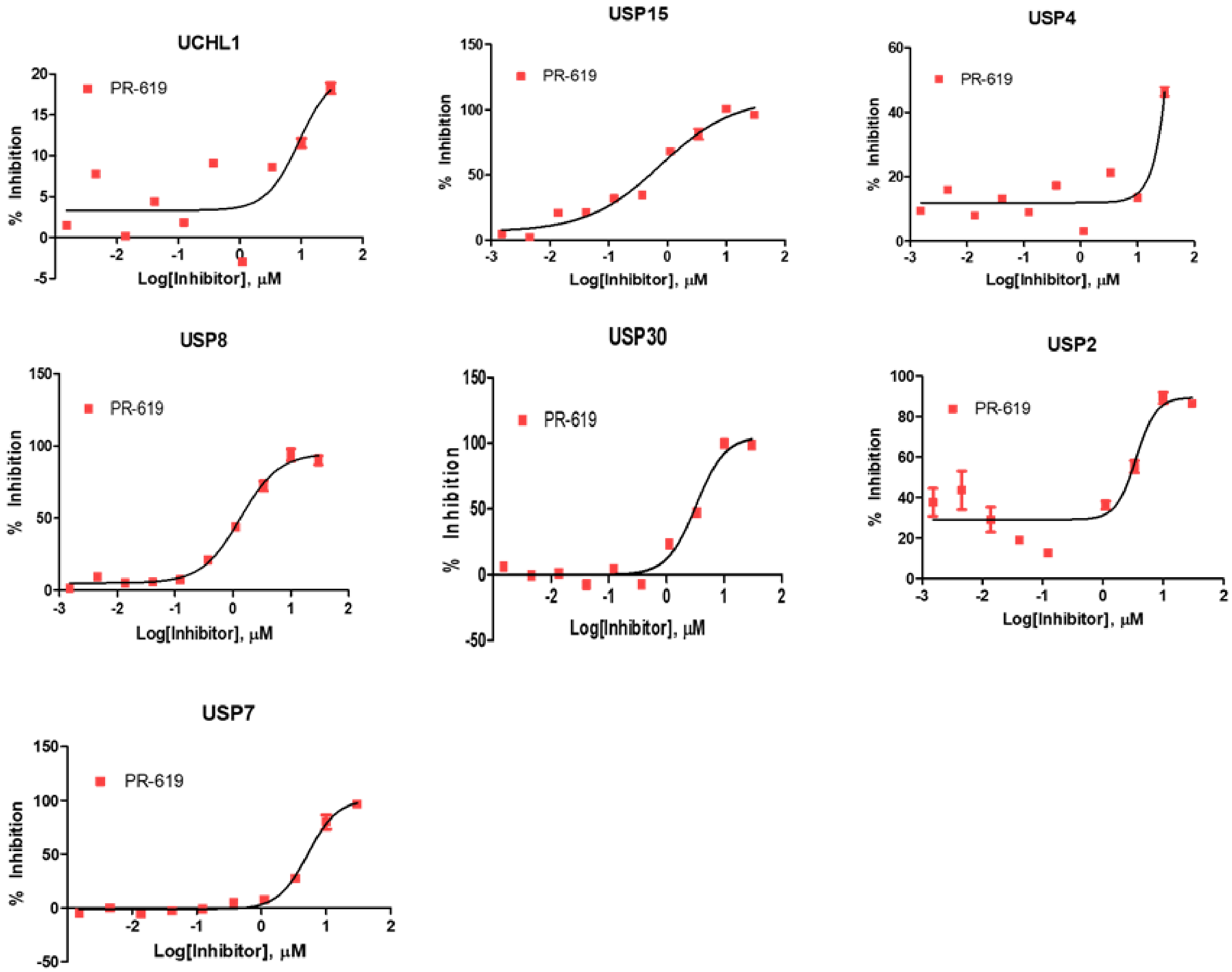

SI. Fig 2: Inhibition curves for PR619 with all the DUBs in the panel as positive control.

All products are for research use only • not intended for human or animal diagnostic or therapeutic uses
Copyright © 2016 LifeSensors, Inc. All Rights Reserved
